# Supplementary material for: Delivery of a BET protein degrader via a CEACAM6-targeted antibody–drug conjugate inhibits tumour growth in pancreatic cancer models
Source: Nat Commun. 2024 Mar 11;15:2192. doi: 10.1038/s41467-024-46167-1 (PMC10928091; doi:10.1038/s41467-024-46167-1)
Supplement: Supplementary file 1 — Supplementary Information [file 41467_2024_46167_MOESM1_ESM.pdf]

Supplementary Information

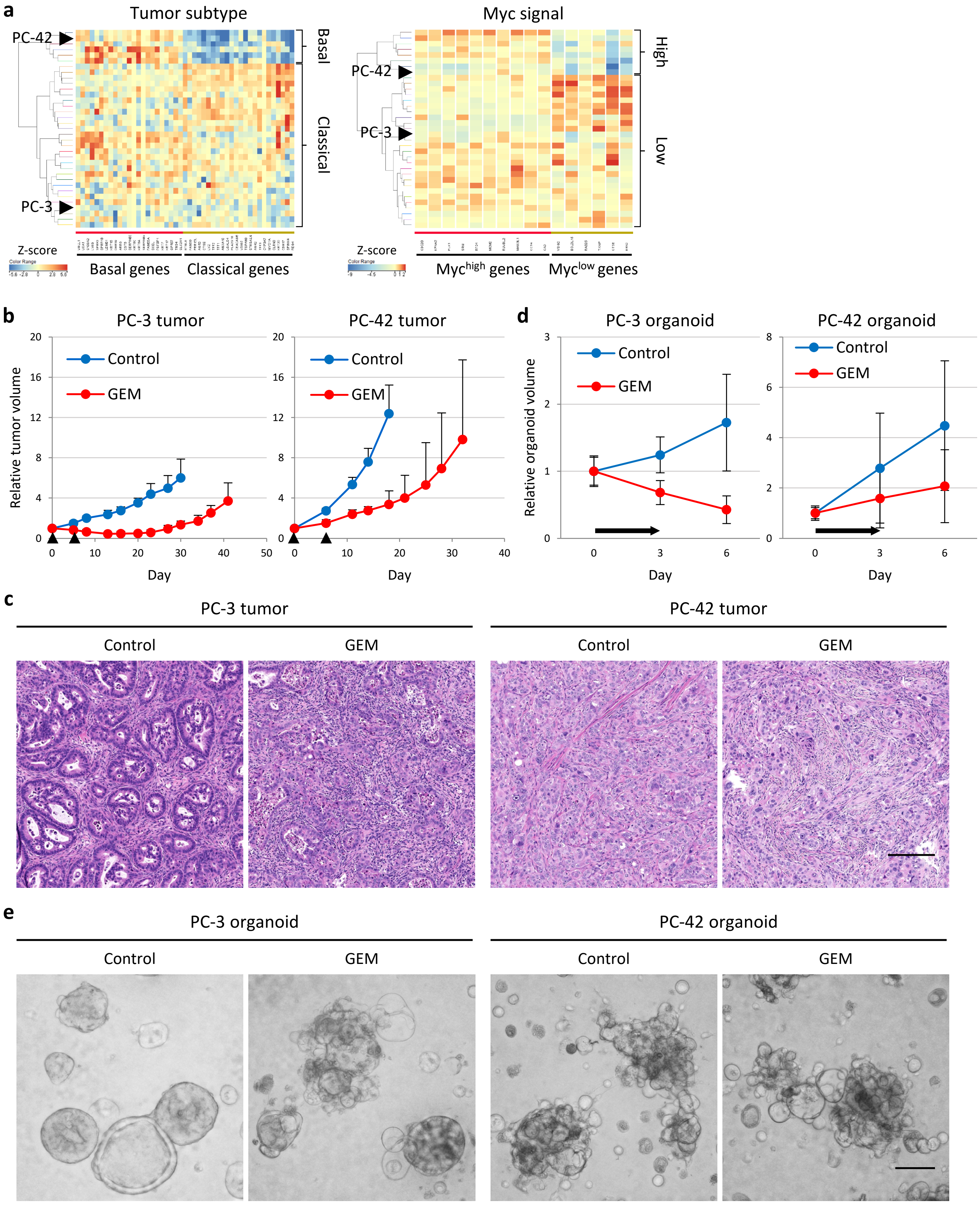

Supplementary Fig. 1 | Characterization of PDAC-PDX tumors and organoids. a, Consensus-clustered heat map of PDAC-PDX dataset. Each set of sample data was clustered into the following subtypes: basal-like vs. classical and Myc signal low vs. Myc signal high. **b,** Tumor growth curves in response to GEM treatment (200 mg/kg) in PC-3 and PC-42 PDAC-PDX models. The average starting tumor volumes were as follows, PC-3: 350 mm^3, PC-42: 180 mm^3. Arrowheads indicate times of drug administration. Data are presented as means ± standard deviation (n = 5 biological replicates). **c,** Hematoxylin and eosin staining of PC-3 and PC-42 tumors with or without GEM treatment (200 mg/kg). Scale bar, 200 µm. **d,** Organoid growth curves in response to GEM treatment (100 nM) in the PC-3 and PC-42 models. Arrows indicate the period of drug treatment. Data are presented as means ± standard deviation (n = 12 biological replicates at minimum). **e,** Organoid images of the PC-3 and PC-42 models with or without GEM treatment (100 nM). Scale bar, 100 µm. **b, d,** Source data are provided as a Source Data file.

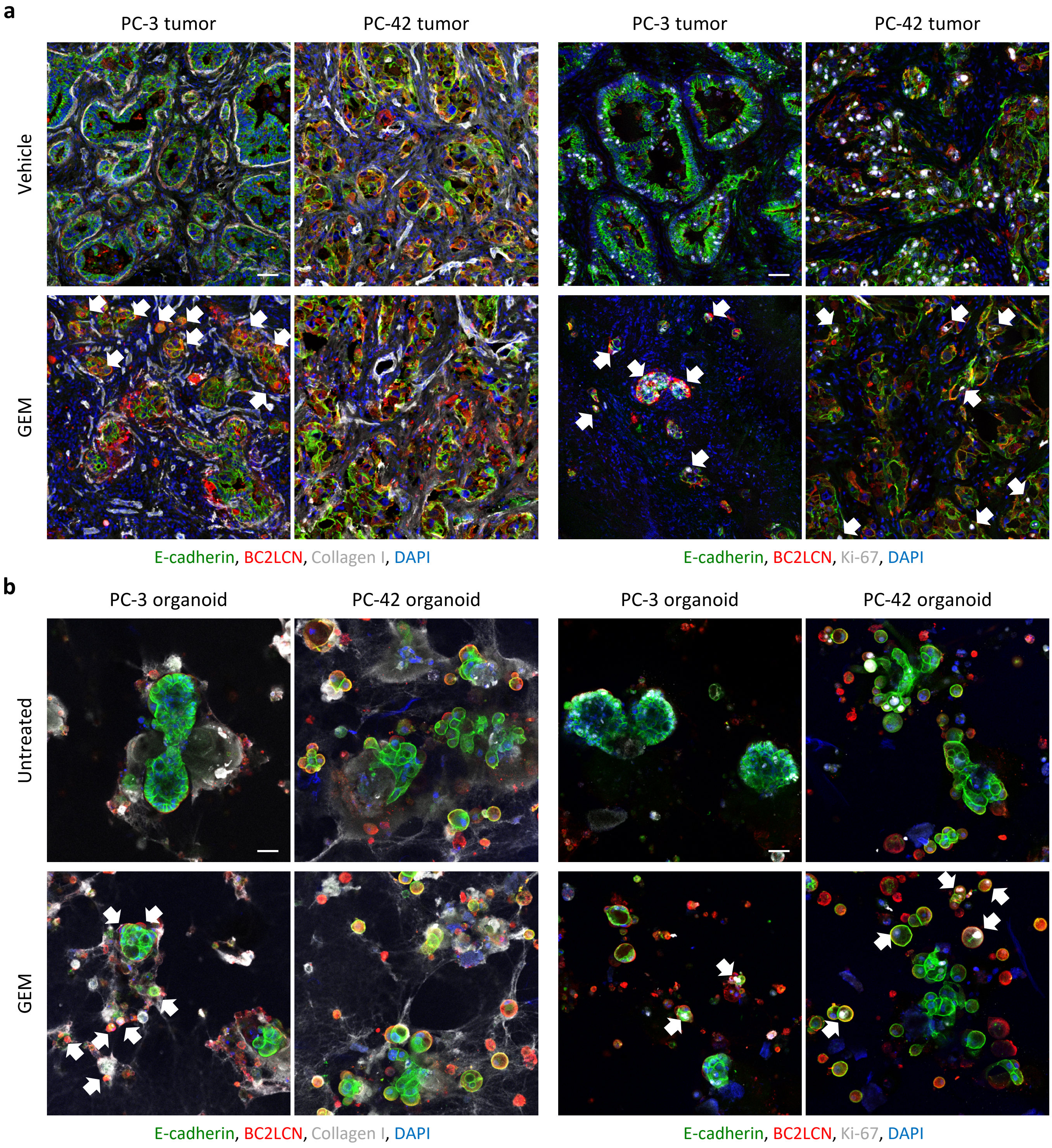

Supplementary Fig. 2 | Organoid culture phenotypically mimics the original PDAC-PDX tumor. a, Immunofluorescence staining for E-cadherin, BC2LCN, collagen I, and Ki-67 in PC-3 and PC-42 tumors with or without GEM treatment (200 mg/kg). Arrows indicate E-cadherin and BC2LCN double-positive cells in the left panels and E-cadherin, BC2LCN, and Ki-67 triple-positive cells in the right panels. Scale bars, 50 µm. **b,** Immunofluorescence staining for E-cadherin, BC2LCN, collagen I, and Ki-67 in PC-3 and PC-42 organoids with or without GEM treatment (100 nM). Arrows indicate E-cadherin and BC2LCN double-positive cells in the left panels and E-cadherin, BC2LCN, and Ki-67 triple-positive cells in the right panels. Scale bars, 50 µm.

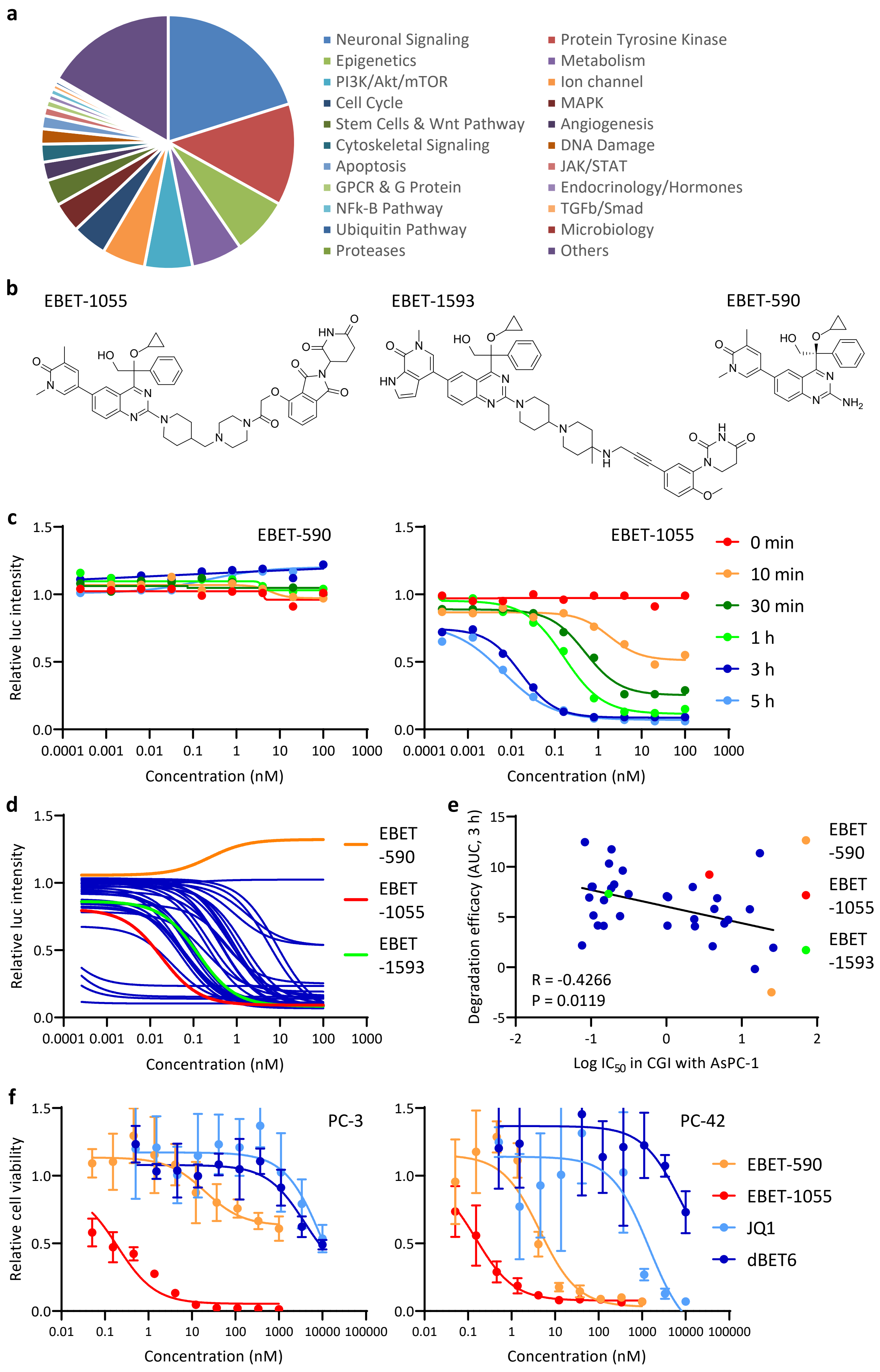
a
Neuronal Signaling
Epigenetics
PI3K/Akt/mTOR
Cell Cycle
Stem Cells & Wnt Pathway
Cytoskeletal Signaling
Apoptosis
GPCR & G Protein
NFk-B Pathway
Ubiquitin Pathway
Proteases
Protein Tyrosine Kinase
Metabolism
Ion channel
MAPK
Angiogenesis
DNA Damage
JAK/STAT
Endocrinology/Hormones
TGFb/Smad
Microbiology
Others
b
EBET-1055
EBET-1593
EBET-590
c
EBET-590
EBET-1055
Relative luc intensity
Concentration (nM)
0 min
10 min
30 min
1 h
3 h
5 h
d
Relative luc intensity
Concentration (nM)
EBET -590
EBET -1055
EBET -1593
e
Degradation efficacy (AUC, 3 h)
R = -0.4266
P = 0.0119
Log IC50 in CGI with AsPC-1
EBET -590
EBET -1055
EBET -1593
f
Relative cell viability
PC-3
PC-42
Concentration (nM)
EBET-590
EBET-1055
JQ1
dBET6

Supplementary Fig. 3 | EBET-1055 compound works as a BET protein degrader and kills PDAC cells. a, Diagram of mechanisms of action of reference compounds in the library. **b,** Chemical structures of EBET-1055, EBET-1593, and EBET-590. **c,** Live-cell monitoring of BET BD1 domain degradation by EBET-590 and EBET-1055 (n = 4 biological replicates). Fitted curves with nonlinear regression are shown as degradation curves. **d,** Degradation curves 3 h after the addition of 35 EBET-derivatives and reference compounds to the live-cell monitoring system (n = 4 biological replicates). **e,** Correlation analysis between degradation efficacy and 50% growth inhibition concentration (IC_{50}) of EBET compounds. Degradation efficacy was measured as the area under the degradation curve (AUC), and IC_{50} was measured in a cell growth inhibition (CGI) assay with AsPC-1 cells. *R*-value and *P*-value calculated by Pearson's correlation and simple linear regression are shown on the scatterplot. **f,** PC-3 and PC-42 organoid growth inhibition by EBET-590, EBET-1055, JQ1, or dBET6. Data are presented as means $\pm$ standard deviation (n = 4 biological replicates). Fitted curves with nonlinear regression are shown. **c-f,** Source data are provided as a Source Data file.

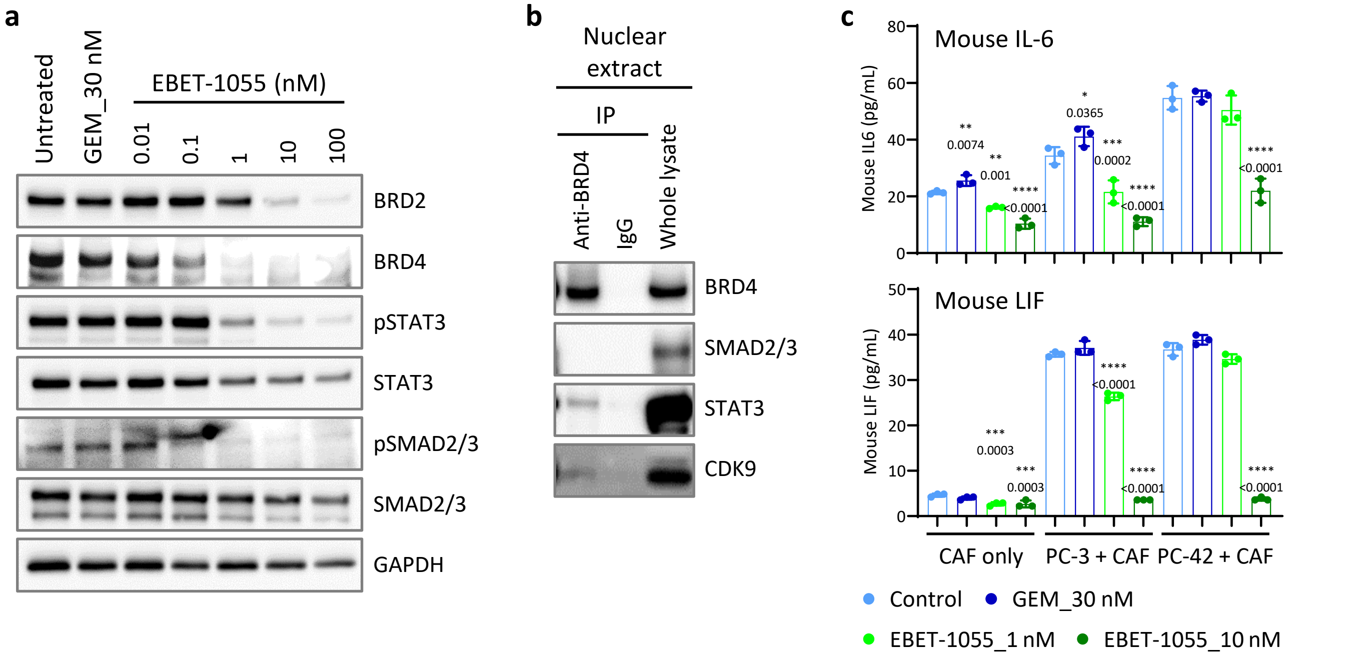

Supplementary Fig. 4 | EBET-1055 compound modulates CAF activity. a, Western blot analysis of BRD2, BRD4, SMAD2/3, and STAT3 in mouse CAFs after treatment with EBET-1055 in 2D culture. **b**, Immunoprecipitation and western blot analysis of BRD4, STAT3, SMAD2/3, and CDK9 in human liver stellate cells. **c,** Quantitation of inflammatory cytokines released from CAFs in co-culture with PC-3 or PC-42 cancer cells. Data are presented as means ± standard deviation (n = 3 biological replicates). *P < 0.05, **P < 0.01, ***P < 0.001, ****P < 0.0001, one-way ANOVA test followed by Dunnett's test between control group and treatment groups in each culture. **a-c,** Source data are provided as a Source Data file.

a

Number of cases	CEACAM6 staining score			Positivity rate
	1+	2+	3+	
50	7 (14%)	16 (32%)	27 (54%)	100%

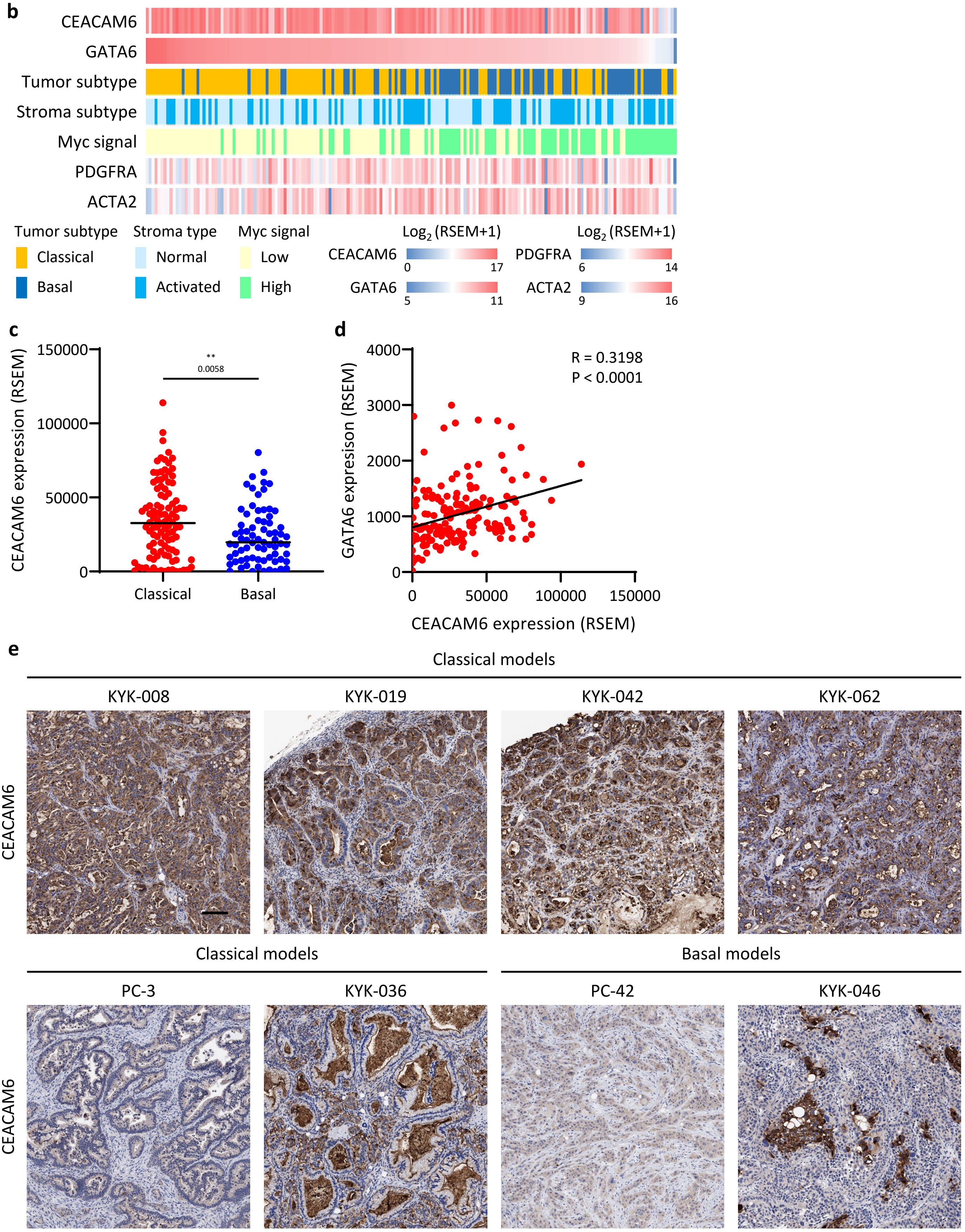

Supplementary Fig. 5 | Expression analysis of PDAC clinical samples, TCGA-PDAC dataset, and PDAC-PDX panel. a, Summary of CEACAM6 staining score and positivity rate with clinical PDAC samples. **b,** Heatmaps showing RNA expression and molecular subtypes of 177 individual PDAC patients from the TCGA dataset. RSEM, RNA-seq by Expectation-Maximization. **c,** *CEACAM6* expression in classical and basal-like PDAC. Bars represent means. **P < 0.01, Mann-Whitney test. **d,** Correlation between *CEACAM6* and *GATA6* expression. R-value and P-value calculated by Pearson's correlation and simple regression line are shown on the scatterplot. **e,** Representative images of CEACAM6 staining of PDX tumors are shown. Scale bar, 100 µm. **c, d,** Source data are provided as a Source Data file.

a

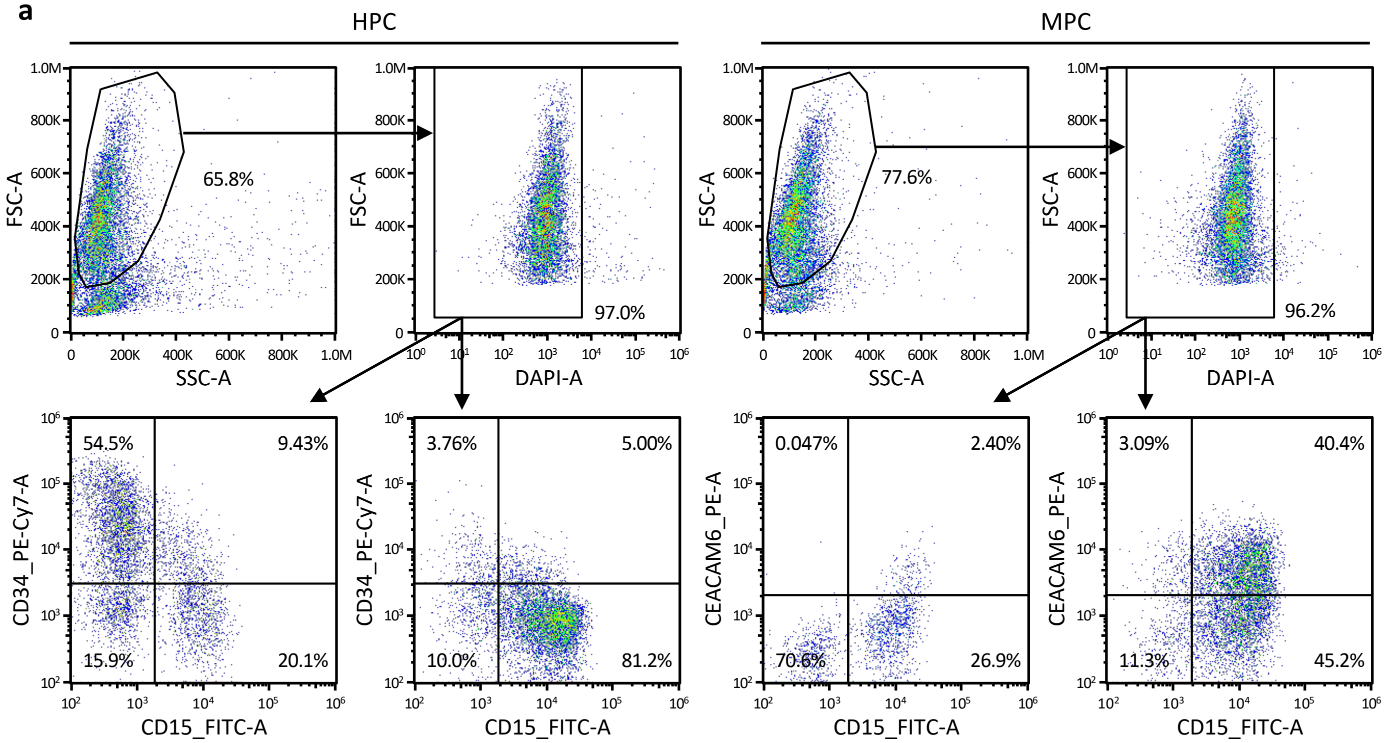

b

	HPAF-II	AsPC-1	MPC	HSAEC	PC-3	PC-42	KYK-008	KYK-054
Number of CEACAM6	791,479	646,033	13,210	384,704	422,361	393,904	1,753,688	803,078

c **d** **e**

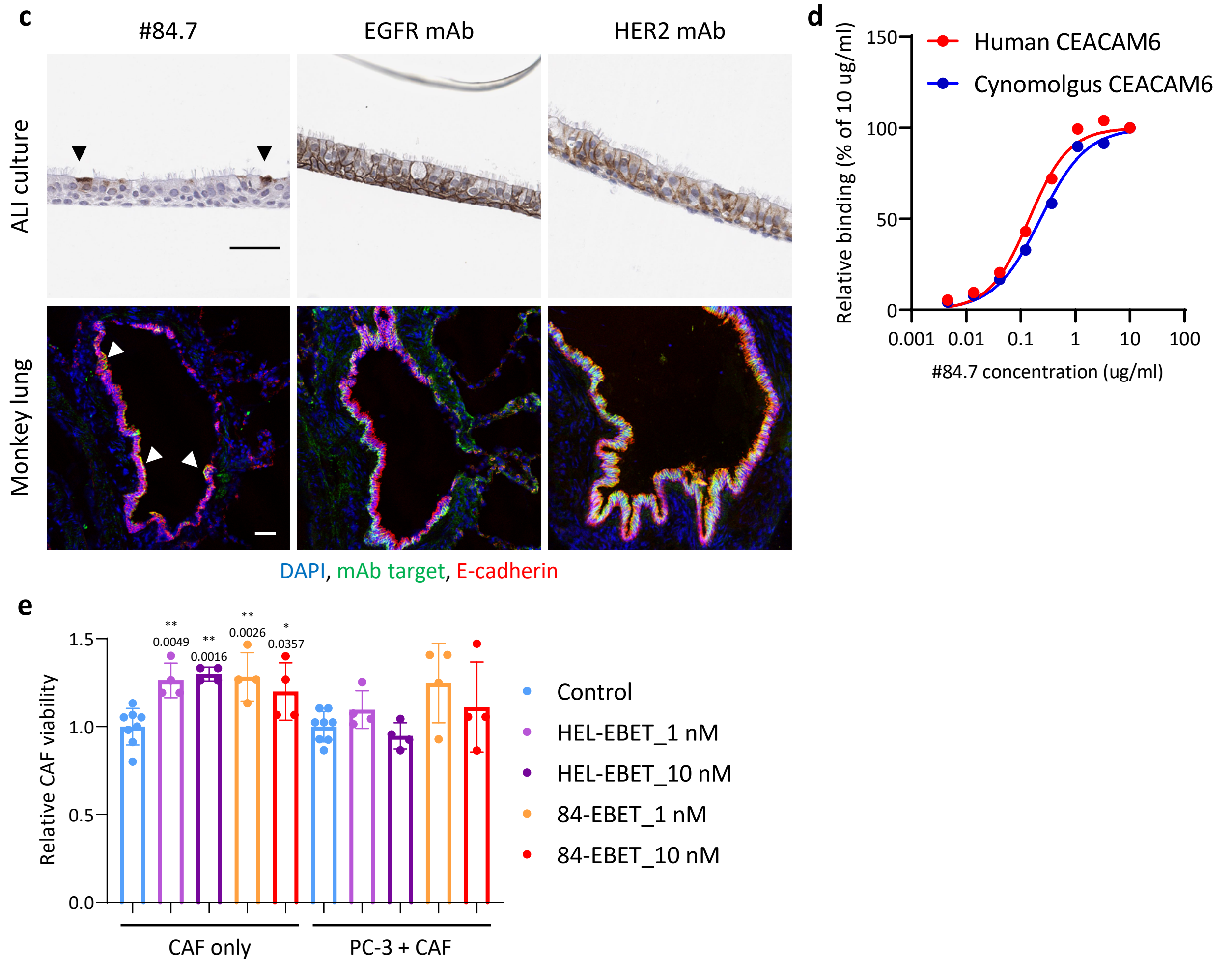

Supplementary Fig. 6 | CEACAM6 is expressed on myeloid progenitor cells and the apical side of the lung epithelium. a, Flow cytometric analysis of CEACAM6 on myeloid progenitor cells (MPCs) differentiated from hematopoietic progenitor cells (HPCs) by using CD15, CD34, and CEACAM6 antibodies. **b,** Absolute quantitation of CEACAM6 on cell surface of human MPCs, HSAECs and PDAC cells. **c,** Immunohistochemical staining of air-liquid interface (ALI) culture of human pulmonary alveolar epithelial cells (upper) and immunofluorescence staining with monkey lung (lower). Arrowheads indicate CEACAM6 staining of the apical side of the lung epithelium. Scale bar, 50 µm. **d,** Flow cytometric analysis using #84.7 antibody of HEK293 cells expressing human or cynomolgus CEACAM6. Binding was quantified by the mean fluorescence intensity of the indicated concentrations. Fitted curves with nonlinear regression are shown. **e,** Cell growth inhibition by 84-EBET in co-culture of PC-3/Fluc and CAF/Rluc cells. The viabilities of each cell were measured by using dual luciferase assay. Data are presented as means $\pm$ standard deviation (n = 4 biological replicates). **d, e,** Source data are provided as a Source Data file.

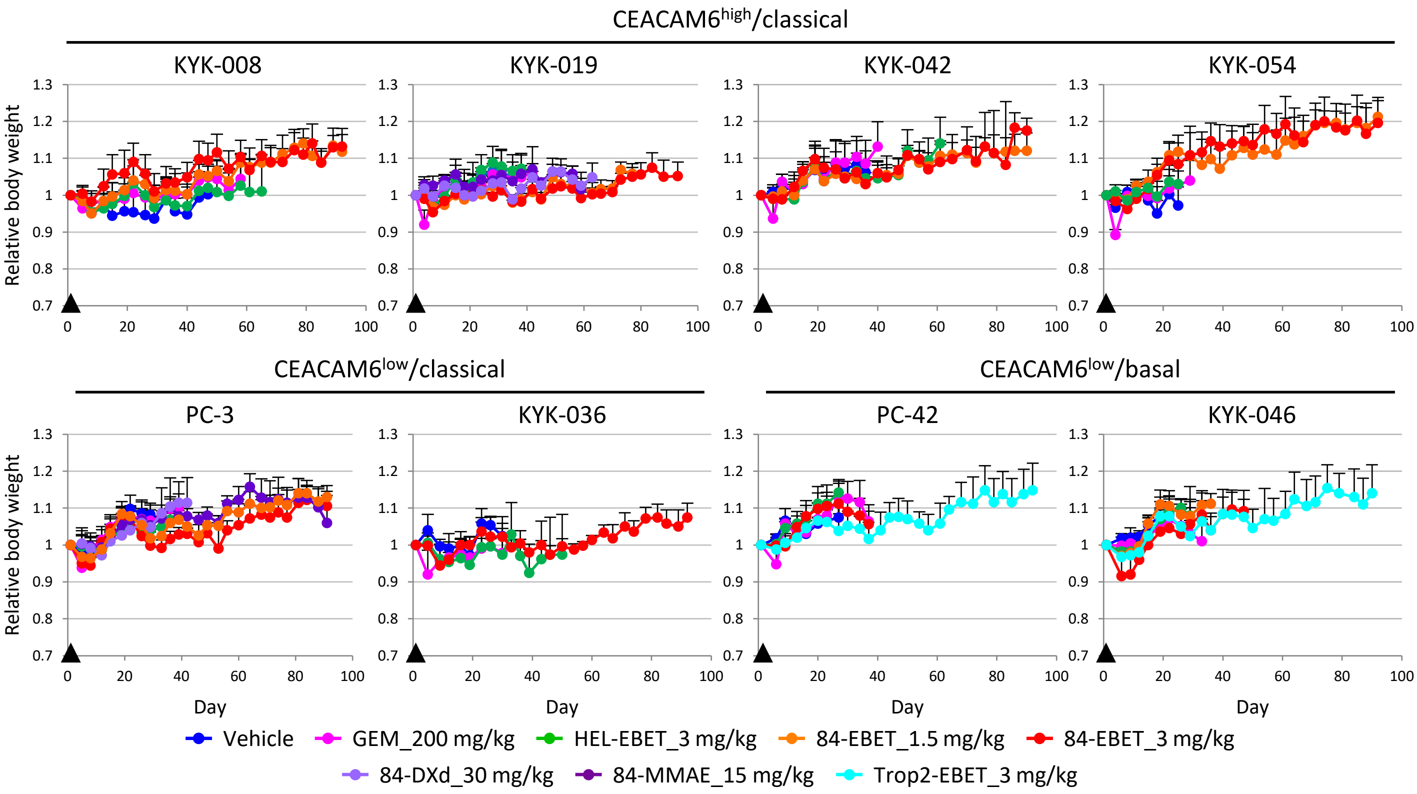

Supplementary Fig. 7 | Effective dose of 84-EBET does not induce body weight loss in mice. Representative body weight curves in response to GEM and ADCs with EBET in the PDAC-PDX panel are shown. Arrowheads indicate times of drug administration. Data are presented as means ± standard deviation (n = 5 mice). Source data are provided as a Source Data file.

a

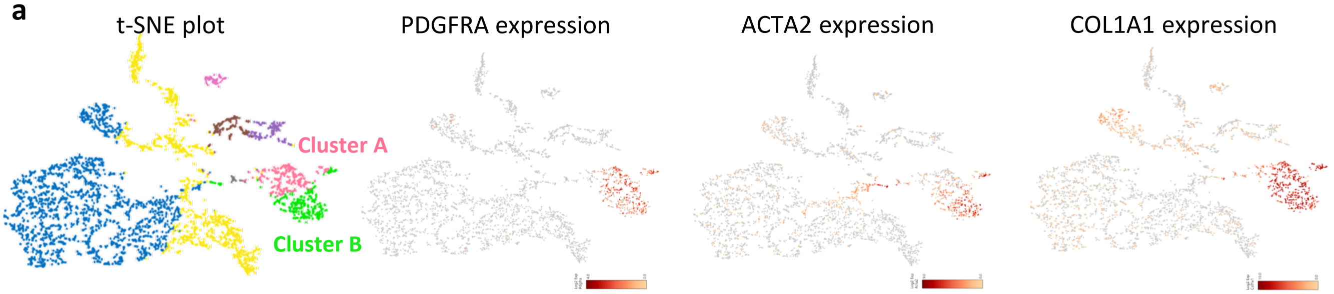

b

Gene set name	ES	NES	NOM p-val	FDR q-val
HALLMARK_TNFA_SIGNALING_VIA_NFKB	0.5982	2.3256	0.0000	0.0000
HALLMARK_IL6_JAK_STAT3_SIGNALING	0.7646	2.2760	0.0000	0.0000
HALLMARK_INFLAMMATORY_RESPONSE	0.5333	1.8883	0.0000	0.0070
HALLMARK_ALLOGRAFT_REJECTION	0.5322	1.8483	0.0000	0.0086
HALLMARK_COMPLEMENT	0.5010	1.8394	0.0029	0.0078
HALLMARK_UV_RESPONSE_UP	0.5192	1.7317	0.0000	0.0205
HALLMARK_INTERFERON_GAMMA_RESPONSE	0.4688	1.6775	0.0031	0.0257
HALLMARK_APOPTOSIS	0.4349	1.5730	0.0091	0.0524
HALLMARK_HYPOXIA	0.4010	1.5307	0.0090	0.0626

c

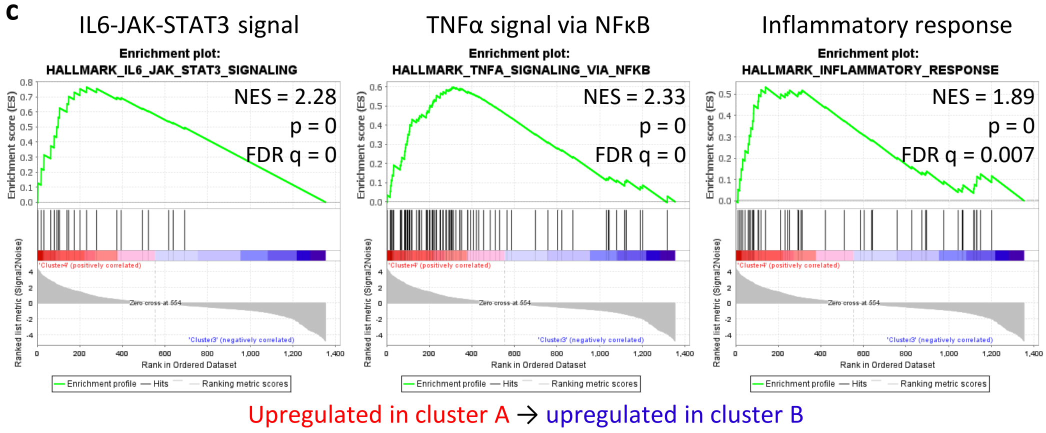

Supplementary Fig. 8 | PDGFRα and α-SMA define inflammatory CAF and myofibroblastic CAF populations, respectively, in PDAC-PDX models. a, Single-cell RNA sequence of stromal cells from PC-3 and PC-42 tumors. A t-distributed stochastic neighbor embedding (t-SNE) map of total stromal cells was color-coded on the basis of clustering. Gene expression gradients of the CAF markers PDGFRA, ACTA2, and COL1A1 are shown on the plots. **b,** Summary of gene set enrichment analysis using genes upregulated in cluster A vs. cluster B. Hallmark gene sets with P-values less than 0.05 are shown. ES, enrichment score; NES, normalize enrichment score; NOM, nominal; FDR, false discovery rate. **c,** Gene set enrichment analysis using genes upregulated in cluster A vs. cluster B. Gene sets: HALLMARK_IL6_JAK_STAT3_SIGNALING, HALLMARK_TNFA_SIGNALING_VIA_NFKB, HALLMARK_INFLAMMATORY_RESPONSE. NESs, P-values, and FDR Q-values are shown on the plots.

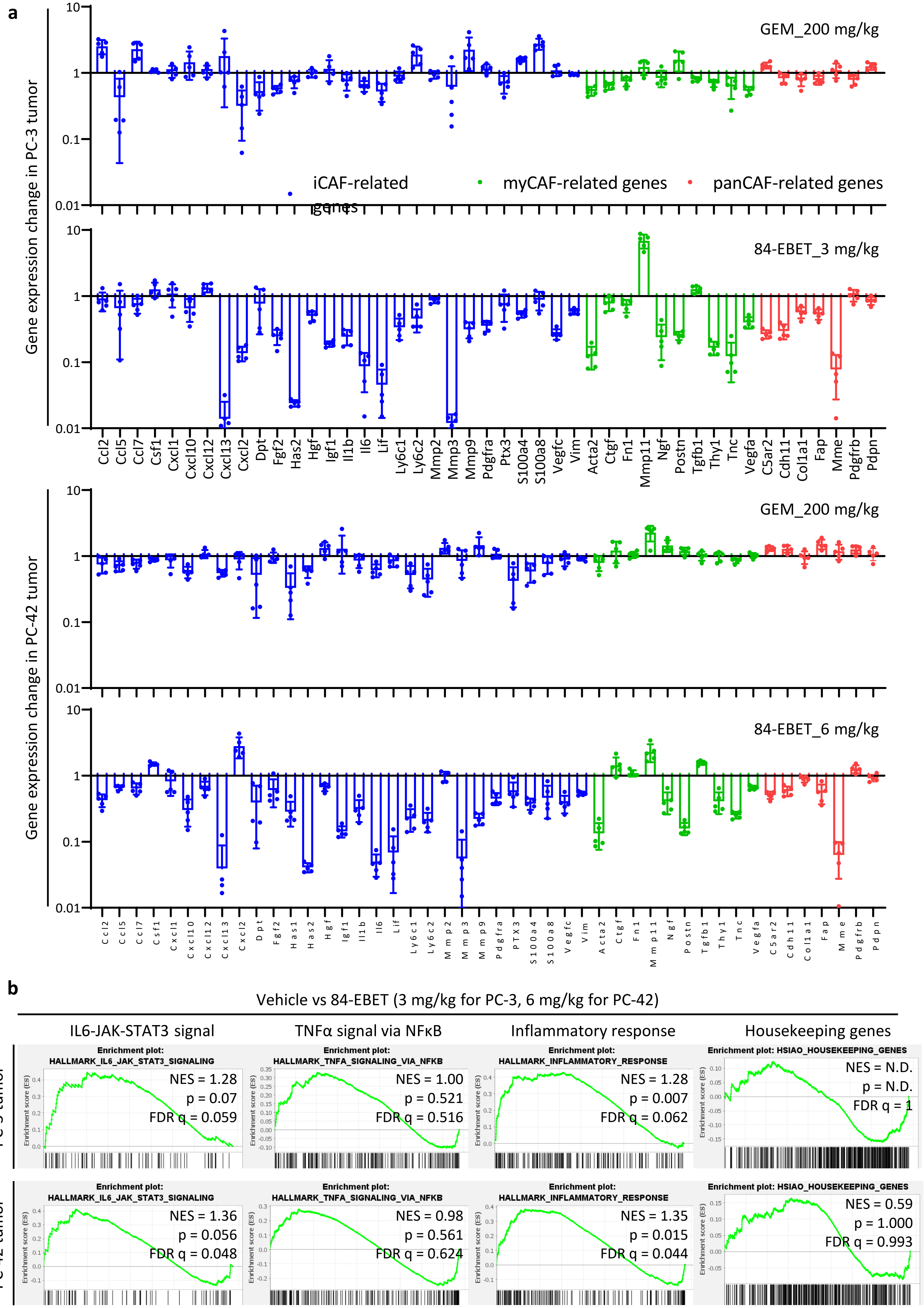
a
GEM_200 mg/kg
84-EBET_3 mg/kg
Gene expression change in PC-3 tumor
iCAF-related genes
myCAF-related genes
panCAF-related genes
10
1
0.1
0.01
Ccl2 Ccl5 Ccl7 Csf1 Cxcl1 Cxcl10 Cxcl12 Cxcl13 Cxcl2 Dpt Fgf2 Has2 Hgf Igf1 Il1b Il6 Lif Ly6c1 Ly6c2 Mmp2 Mmp3 Mmp9 Pdgfra Ptx3 S100a4 S100a8 Vegfc Vim Acta2 Ctgf Fn1 Mmp11 Ngf Postn Tgfb1 Thy1 Tnc Vegfa C5ar2 Cdh11 Col1a1 Fap Mme Pdgfrb Pdpn
GEM_200 mg/kg
84-EBET_6 mg/kg
Gene expression change in PC-42 tumor
Ccl2 Ccl5 Ccl7 Csf1 Cxcl1 Cxcl10 Cxcl12 Cxcl13 Cxcl2 Dpt Fgf2 Has1 Has2 Hgf Igf1 Il1b Il6 Lif Ly6c1 Ly6c2 Mmp2 Mmp3 Mmp9 Pdgfra PTX3 S100a4 S100a8 Vegfc Vim Acta2 Ctgf Fn1 Mmp11 Ngf Postn Tgfb1 Thy1 Tnc Vegfa C5ar2 Cdh11 Col1a1 Fap Mme Pdgfrb Pdpn
b
Vehicle vs 84-EBET (3 mg/kg for PC-3, 6 mg/kg for PC-42)
IL6-JAK-STAT3 signal
TNFα signal via NFκB
Inflammatory response
Housekeeping genes
PC-3 tumor
Enrichment plot: HALLMARK_IL6_JAK_STAT3_SIGNALING
NES = 1.28
p = 0.07
FDR q = 0.059
Enrichment plot: HALLMARK_TNFA_SIGNALING_VIA_NFKB
NES = 1.00
p = 0.521
FDR q = 0.516
Enrichment plot: HALLMARK_INFLAMMATORY_RESPONSE
NES = 1.28
p = 0.007
FDR q = 0.062
Enrichment plot: HSIAO_HOUSEKEEPING_GENES
NES = N.D.
p = N.D.
FDR q = 1
PC-42 tumor
Enrichment plot: HALLMARK_IL6_JAK_STAT3_SIGNALING
NES = 1.36
p = 0.056
FDR q = 0.048
Enrichment plot: HALLMARK_TNFA_SIGNALING_VIA_NFKB
NES = 0.98
p = 0.561
FDR q = 0.624
Enrichment plot: HALLMARK_INFLAMMATORY_RESPONSE
NES = 1.35
p = 0.015
FDR q = 0.044
Enrichment plot: HSIAO_HOUSEKEEPING_GENES
NES = 0.59
p = 1.000
FDR q = 0.993
Enrichment score (ES)

Supplementary Fig. 9 | 84-EBET modulates stromal cells and their signaling via the bystander effect. a, Expression change of CAF-related genes in the stroma of PC-3 and PC-42 tumors upon GEM or 84-EBET treatment is shown. Data are presented as means ± standard deviation (n = 5 mice). **b,** Gene set enrichment analysis using upregulated and downregulated stromal genes by 84-EBET treatment is shown. Gene sets: HALLMARK_IL6_JAK_STAT3_SIGNALING, HALLMARK_TNFA_SIGNALING_VIA_NFKB, HALLMARK_INFLAMMATORY_RESPONSE, HSIAO_HOUSEKEEPING_GENES. NESs, *P*-values, and FDR *Q*-values are shown on the plots. **a,** Source data are provided as a Source Data file.

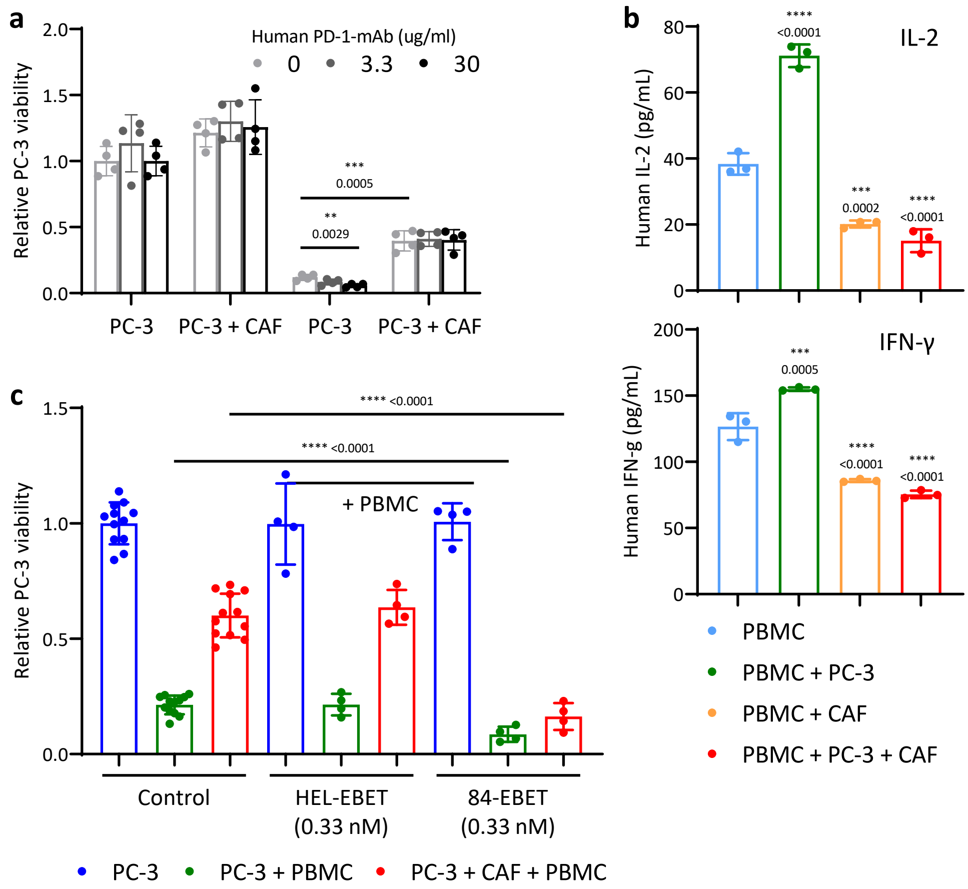

Supplementary Fig. 10 | 84-EBET facilitates cancer-cell killing by T cells in tri-culture with CAFs. a, Assay of killing of PC-3/Fluc cancer cells by human PBMCs in co-culture with mouse CAFs. The viability of PC-3 cancer cells was measured by Fluc reporter assay. Anti-human PD-1 antibody was added at the indicated concentrations. Data are presented as means ± standard deviation (n = 4 biological replicates). **P < 0.01, one-way ANOVA test followed by Dunnett's test. ***P < 0.001, unpaired t-test. **b,** Quantitation of released human IL-2 and IFN-γ in tri-culture of PC-3 cancer cells, hPBMCs, and CAFs (n = 3). Data are presented as means ± standard deviation (n = 3 biological replicates). ***P < 0.001, ****P < 0.0001, one-way ANOVA test followed by Dunnett's test between hPBMC monoculture and co-cultures. **c,** Assay of killing of PDAC PC-3/Fluc cells by hPBMCs in co-culture with mouse CAFs in the presence of 84-EBET. The viability of PC-3 cancer cells was measured by Fluc reporter assay. Data are presented as means ± standard deviation (n = 4 biological replicates). ****P < 0.0001, one-way ANOVA test followed by Dunnett's test. **a-c,** Source data are provided as a Source Data file.

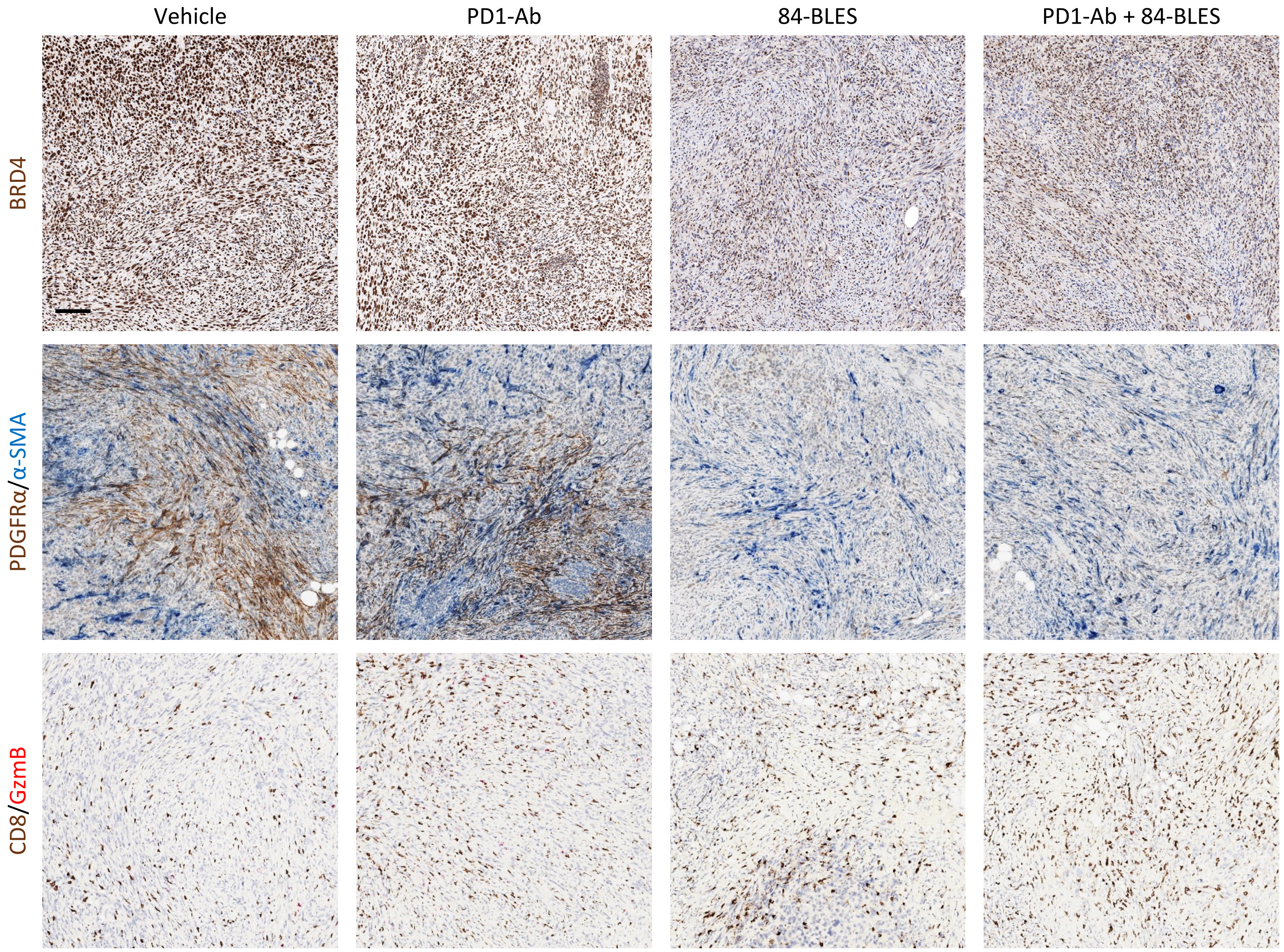

Supplementary Fig. 11 | 84-EBET modulates CAFs and increases activated T cells in tumors. Immunohistochemical staining for BRD4, PDGFRα, α-SMA, CD8, and granzyme B (GzmB) in the Pan02/hCEACAM6 tumors 3 and 7 days after drug treatment. Scale bar, 100 µm.

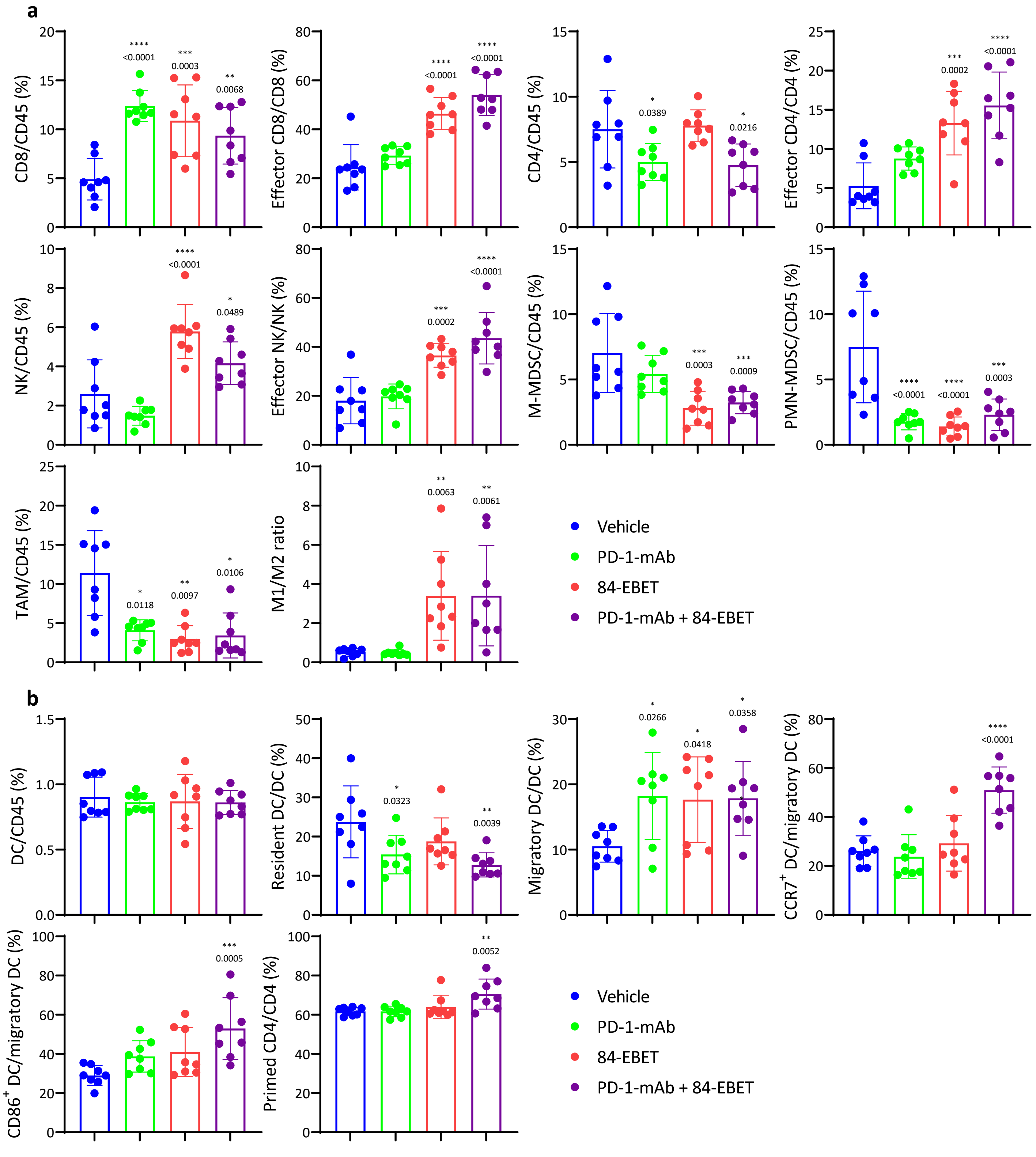

Supplementary Fig. 12 | 84-EBET improves immunosuppressive tumor microenvironment. a, Immune profiling of Pan02/hCEACAM6 tumors 7 days after drug treatment by mass cytometry. The markers for each immune cell are as follows: IFNγ$^+$CD8$^+$ for effector CD8 cells, IFNγ$^+$CD4$^+$ for effector CD4 cells, CD335$^+$ for NK cells, IFNγ$^+$CD335$^+$ for effector NK cells, Arg1$^+$Ly6c$^+$CD11b$^+$ Ly6g$^-$ for monocytic myeloid-derived suppressor cells (M-MDSCs), Arg1$^+$Ly6g$^+$CD11b$^+$ Ly6c$^-$ for polymorphonuclear myeloid-derived suppressor cells (PMN-MDSCs), CD11b$^+$F4/80$^+$ for tumor-associated macrophages (TAMs), CD86$^+$iNOS$^+$CD11b$^+$F4/80$^+$ for M1-type TAMs, CD206$^+$Arg1$^+$CD11b$^+$F4/80$^+$ for M2-type TAMs. Data are presented as means ± standard deviation (n = 8 mice). *$P < 0.05$, **$P < 0.01$, ***$P < 0.001$, ****$P < 0.0001$, one-way ANOVA test followed by Dunnett's test between vehicle-treated group and drug-treated groups. **b,** Immune profiling of tumor-draining lymph nodes of mice with Pan02/hCEACAM6 tumors 7 days after drug treatment by mass cytometry. The markers for each immune cell are as follows: MHCII$^+$CD11c$^+$ for dendritic cells (DCs), MHCII$^+$CD11c^{med} for resident DCs, MHCII$^+$CD11c^{high} for migratory DCs, CCR7$^+$MHCII$^+$CD11c^{high} or CD86$^+$MHCII$^+$CD11c^{high} for mature DCs, IL2$^+$CD4$^+$ for primed CD4 cells. Data are presented as means ± standard deviation (n = 8 mice). *$P < 0.05$, **$P < 0.01$, ***$P < 0.001$, ****$P < 0.0001$, one-way ANOVA test followed by Dunnett's test between vehicle-treated group and drug-treated groups. **a, b,** Source data are provided as a Source Data file.

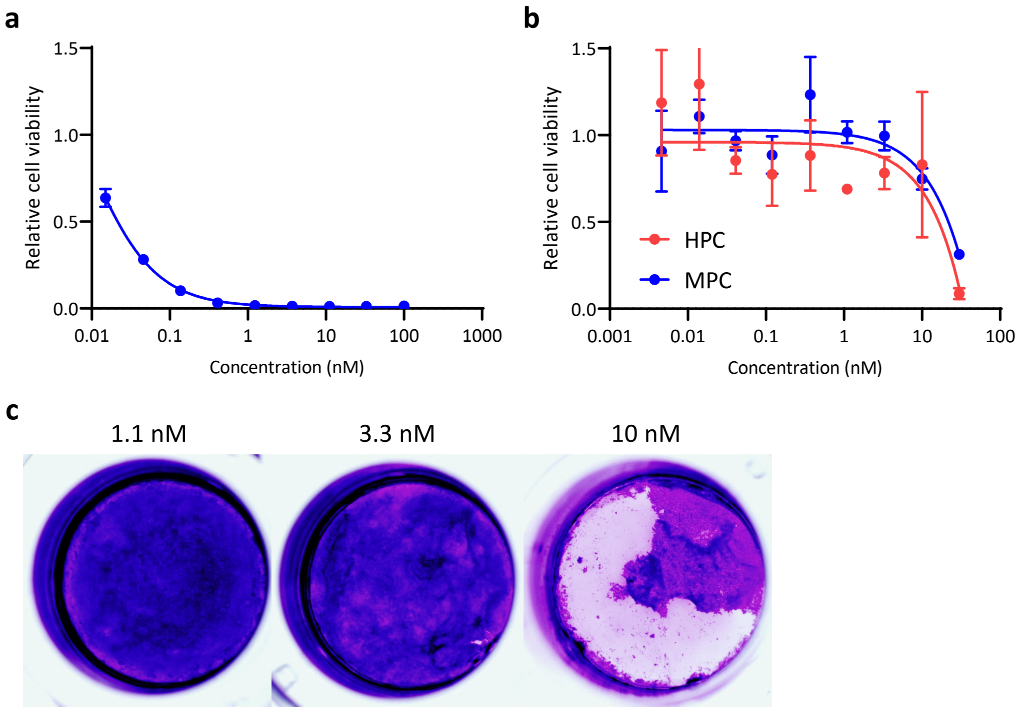

Supplementary Fig. 13 | In vitro profiling of the final candidate of 84-EBET. a, Cell growth inhibition by 84-EBET with HPAF-II cells. Data are presented as means ± standard deviation (n = 4 biological replicates). Fitted curves with nonlinear regression are shown. **b,** Cell growth inhibition by 84-EBET with human hematopoietic progenitor cells (HPCs) and myeloid progenitor cells (MPCs). Data are presented as means ± standard deviation (n = 4 biological replicates). Fitted curves with nonlinear regression are shown. **c,** In vitro lung toxicity assay with air-liquid interface (ALI) culture of human lung epithelial cells. Five days after incubation of 84-EBET in the lower chamber of ALI culture, surviving epithelial cells were stained with crystal violet. **a, b,** Source data are provided as a Source Data file.

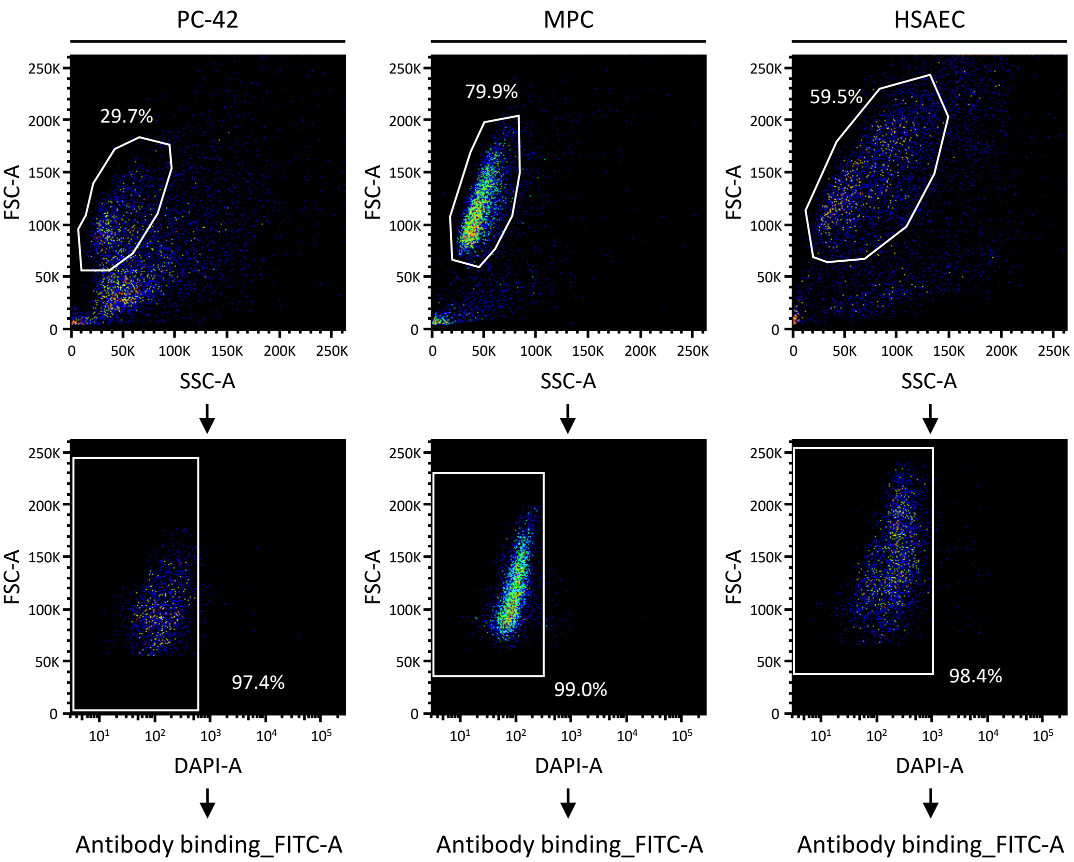

Supplementary Fig. 14 | Gating strategy of flow cytometry in Fig 2d.

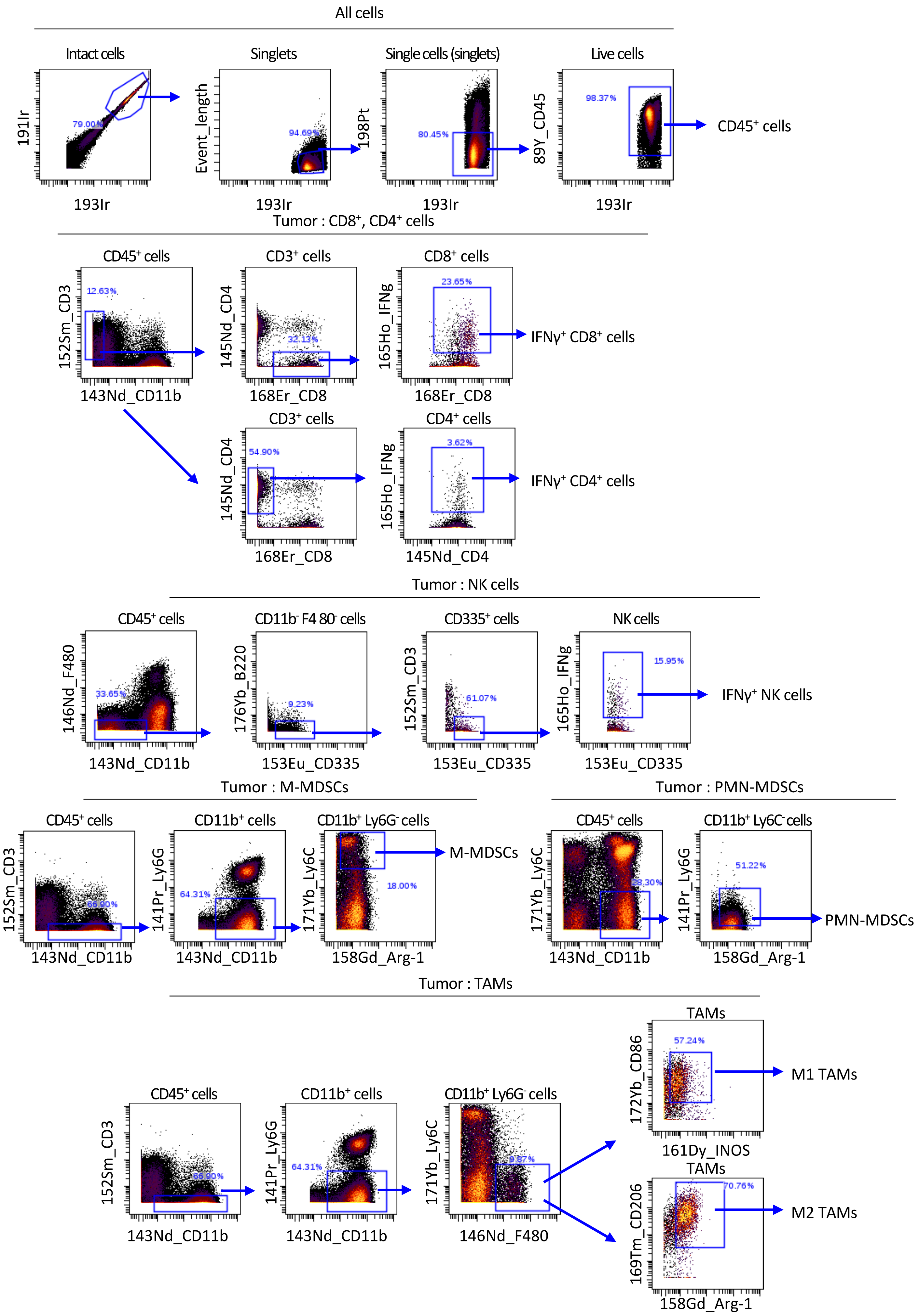

Supplementary Fig. 15 | Gating strategy of mass cytometry in Supplementary Fig 12a.

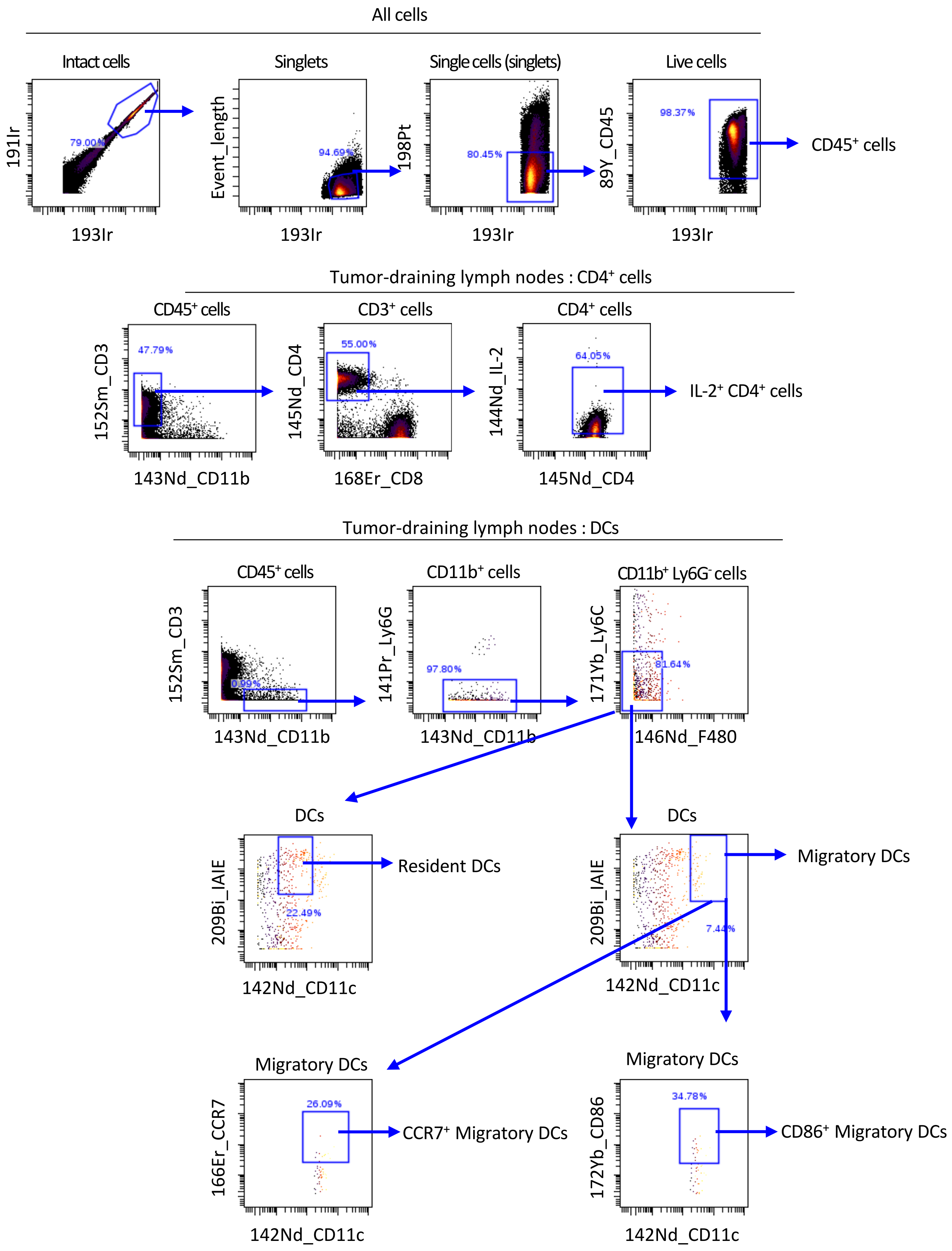

Supplementary Fig. 16 | Gating strategy of mass cytometry in Supplementary Fig 12b.

	Company	Product ID	Target	Clone	Dilution	Label	Product type
**Surface markers**							
1	Fluidigm	3089005B	CD45	30-F11	1:50	89Y	Catalog Antibodies
2	Fluidigm	3143015B	CD11b (Mac-1)	M1/70	1:100	143Nd	Catalog Antibodies
3	Fluidigm	3141008B	Ly-6G	1A8	1:100	141Pr	Catalog Antibodies
4	Fluidigm	201171A	Ly-6C		1:100	171Yb	Labeling Kit
5	Fluidigm	3209006B	I-A/I-E	M5/114.15.2	1:167	209Bi	Catalog Antibodies
6	Fluidigm	3146008B	F4/80	BM8	1:50	146Nd	Catalog Antibodies
7	Fluidigm	3172016B	CD86	GL1	1:50	172Yb	Catalog Antibodies
8	Fluidigm	3169021B	CD206 (MMR)	C068C2	1:100	169Tm	Catalog Antibodies
9	Fluidigm	3142003B	CD11c	N418	1:50	142Nd	Catalog Antibodies
10	Fluidigm	201166A	CD197 (CCR7)		1:50	166Er	Labeling Kit
11	Fluidigm	3153006B	CD335 (NKp46)	29A1.4	1:50	153Eu	Catalog Antibodies
12	Fluidigm	3152004B	CD3e	145-2C11	1:50	152Sm	Catalog Antibodies
13	Fluidigm	3145002B	CD4	RM4-5	1:50	145Nd	Catalog Antibodies
14	Fluidigm	3168003B	CD8a	53-6.7	1:50	168Er	Catalog Antibodies
**Intracellular markers**							
15	Fluidigm	3165003B	IFNg	XMG1.2	1:50	165Ho	Catalog Antibodies
16	Fluidigm	201158A	Arginase-1		1:100	158Gd	Labeling Kit
17	Fluidigm	3161011B	iNOS (NOS2)	CXNFT	1:50	161Dy	Catalog Antibodies
18	Fluidigm	3144002B	IL-2	JES6-5H4	1:50	144Nd	Catalog Antibodies

**Antibody kit for labeling**				
**Company**	**Product ID**	**Target**	**Clone**	**Dilution**
Novus Biologicals	NBP1-28046	Ly-6C Antibody	HK1.4	Prediluted
Sony Biotechnology	1200505	Anti-mouse CD197 (CCR7)	4B12	Prediluted
GENETEX	GTX634218	Arginase 1 antibody	GT5811	Prediluted

Supplementary Table 1 | Antibody list for mass cytometry.
