# Supplementary material for: Delivery of a BET protein degrader via a CEACAM6-targeted antibody–drug conjugate inhibits tumour growth in pancreatic cancer models
Source: Nat Commun. 2024 Mar 11;15:2192. doi: 10.1038/s41467-024-46167-1 (PMC10928091; doi:10.1038/s41467-024-46167-1)
Supplement: Supplementary file 3 — Reporting Summary [file 41467_2024_46167_MOESM3_ESM.pdf]

nature portfolio

Corresponding author(s): Youya Nakazawa

Last updated by author(s): Nov 24, 2023

Reporting Summary

Nature Portfolio wishes to improve the reproducibility of the work that we publish. This form provides structure for consistency and transparency in reporting. For further information on Nature Portfolio policies, see our Editorial Policies and the Editorial Policy Checklist.

Statistics

For all statistical analyses, confirm that the following items are present in the figure legend, table legend, main text, or Methods section.

n/a	Confirmed	
☐	☒	The exact sample size (*n*) for each experimental group/condition, given as a discrete number and unit of measurement
☐	☒	A statement on whether measurements were taken from distinct samples or whether the same sample was measured repeatedly
☐	☒	The statistical test(s) used AND whether they are one- or two-sided<br>*Only common tests should be described solely by name; describe more complex techniques in the Methods section.*
☒	☐	A description of all covariates tested
☒	☐	A description of any assumptions or corrections, such as tests of normality and adjustment for multiple comparisons
☐	☒	A full description of the statistical parameters including central tendency (e.g. means) or other basic estimates (e.g. regression coefficient) AND variation (e.g. standard deviation) or associated estimates of uncertainty (e.g. confidence intervals)
☐	☒	For null hypothesis testing, the test statistic (e.g. *F*, *t*, *r*) with confidence intervals, effect sizes, degrees of freedom and *P* value noted<br>*Give P values as exact values whenever suitable.*
☒	☐	For Bayesian analysis, information on the choice of priors and Markov chain Monte Carlo settings
☒	☐	For hierarchical and complex designs, identification of the appropriate level for tests and full reporting of outcomes
☐	☒	Estimates of effect sizes (e.g. Cohen's *d*, Pearson's *r*), indicating how they were calculated

Our web collection on statistics for biologists contains articles on many of the points above.

Software and code

Policy information about availability of computer code

Data collection	Envision microplate reader system: luminescence data in CellTiter-Glo and luciferase assays<br>GEPIA server: TCGA and GTEx data<br>UCSC Xena Data Hubs: TCGA data<br>NovaSeq system: RNA sequencing<br>Hiseq X system: single-cell RNA sequencing<br>LSRFortessa flow cytometry system: flow cytometry data<br>Helios a CyTOF system: mass cytometry data<br>TCS SP8 confocal microscope system: confocal microscopy of IF samples<br>Aperio AT2 slide scanner system: microscopy of IHC samples<br>BZ-810 microscope system: microscopy of ALI culture samples<br>IVIS spectrum in vivo imaging system: luminescence data in mouse xenograft assays
Data analysis	GEPIA server: gene expression analysis using TCGA and GTEx data<br>Cell Ranger software (v3.0.2), Loupe Browser (v5.1.0): analysis of single-cell RNA sequence data<br>Strand NGS (v3.4): hierarchical clustering with RNA sequence data<br>GSEA software (v4.1.0), MSigDB (v5.2): gene-set enrichment analysis<br>FlowJo software (v10.8.1): flow cytometry analysis<br>Cytobank Premium (v10.1): mass cytometry analysis<br>Leica Application Suite X software (v3.7.4), Image J (v1.8.0): processing of IF images

HALO image analysis software (v2.3.2089.69): quantitation of IHC images
GraphPad Prism software (v9.4.1): graphing and statistical analysis

For manuscripts utilizing custom algorithms or software that are central to the research but not yet described in published literature, software must be made available to editors and reviewers. We strongly encourage code deposition in a community repository (e.g. GitHub). See the Nature Portfolio guidelines for submitting code & software for further information.

Data

Policy information about availability of data

All manuscripts must include a data availability statement. This statement should provide the following information, where applicable:

- Accession codes, unique identifiers, or web links for publicly available datasets
- A description of any restrictions on data availability
- For clinical datasets or third party data, please ensure that the statement adheres to our policy

The RNA sequencing data generated for this study are available at the European Genome-phenome Archive (https://ega-archive.org/) with study IDs EGAS00001007070 and EGAS00001007212. The RNA sequencing data of TCGA used in this study is available at the UCSC Xena Data Hubs with dataset ID TCGA.PAAD.sampleMap/HiSeqV2. Synthesis and characterization information of the compounds used in this study is available in patents with application number: 63/373646, 63/377518 and WO numbers: WO2009099741, WO2015095223, WO2019044947, WO2003043583.

Human research participants

Policy information about studies involving human research participants and Sex and Gender in Research.

Reporting on sex and gender	This study did not involve human research.
Population characteristics	This study did not involve human research.
Recruitment	This study did not involve human research.
Ethics oversight	This study did not involve human research.

Note that full information on the approval of the study protocol must also be provided in the manuscript.

Field-specific reporting

Please select the one below that is the best fit for your research. If you are not sure, read the appropriate sections before making your selection.

☒ Life sciences ☐ Behavioural & social sciences ☐ Ecological, evolutionary & environmental sciences

For a reference copy of the document with all sections, see nature.com/documents/nr-reporting-summary-flat.pdf

Life sciences study design

All studies must disclose on these points even when the disclosure is negative.

Sample size	No statistic method was used to predetermine sample size in mouse xenograft assays. Sample sizes were selected to be adequate based on the magnitude and consistency of measurable differences between groups in preliminary experiments.
Data exclusions	No data was excluded.
Replication	All attempts at replication were successful.To ensure reproducibility of efficacy of CEACAM6-EBET-ADC, in vitro and in vivo efficacy assays were performed using 16 PDAC-organoid models, 16 PDAC-PDX models, 3 PDAC-CDX models and 2 PDAC cell lines, with no inconsistent data between models or assays. For representative models, such as PC-3, PC-42, KYK-017, KYK-019, KYK-065, KYK-069, HPAF-II, AsPC-1, and Pan02 models, up to 200 analogues of CEACAM6-EBET-ADC were also evaluated. There was no discrepancy in efficacy among the analogues, and the degree of efficacy depended on the target degradation efficiency of the analogue. In this article, data of lead ADC, 84-EBET-1593, are shown.
Randomization	For in vivo studies, mice and monkeys were randomly assigned to each treatment group. For in vitro studies, samples were randomly assigned to each treatment group.
Blinding	Data collection and analysis were not performed with blinding to the conditions of the experiments. All the data analysis is based on absolute quantification by instrumentation and does not include subjective factors. For IHC and IF analysis, the representative images were selected from multiple images or the entire image of the samples, with minimal noise or blurring, that could be representative of the entire samples.

Reporting for specific materials, systems and methods

We require information from authors about some types of materials, experimental systems and methods used in many studies. Here, indicate whether each material, system or method listed is relevant to your study. If you are not sure if a list item applies to your research, read the appropriate section before selecting a response.

Materials & experimental systems

n/a	Involved in the study
☐	☒ Antibodies
☐	☒ Eukaryotic cell lines
☒	☐ Palaeontology and archaeology
☐	☒ Animals and other organisms
☒	☐ Clinical data
☒	☐ Dual use research of concern

Methods

n/a	Involved in the study
☒	☐ ChIP-seq
☐	☒ Flow cytometry
☒	☐ MRI-based neuroimaging

Antibodies

Antibodies used

*ADC prep:
EGFR (cetuximab, Merck, 5 mg/mL)
HER2 (trastuzumab, Chugai, 21 mg/mL)
Trop-2 (sacituzumab, purified by EPAT, 5 mg/mL)
HEL3 (purified by KAN, developed internally, 5 mg/mL)
CEACAM6 (#84.7, developed internally, 5 mg/mL)

*Mouse xenograft assay:
InVivoMAb anti-mouse PD-1 (CD279) (BE0146, BioXCell)

*Monkey biodistribution assay:
EGFR (cetuximab, Merck, 0.75 mg/mL, 7.5 mg/kg)
HER2 (trastuzumab, Chugai, 3.75 mg/mL, 7.5 mg/kg)
HEL3 (purified by KAN, developed internally, 3.75 mg/mL, 7.5 mg/kg)
CEACAM6 (#84.7, developed internally, 3.75 mg/mL, 7.5 mg/kg)

*Flow cytometry assay:
CEACAM6 (#84.7, developed internally, 10 µg/mL; D028-3, MBL, 1:100)
Mouse H-2Kd/H-2Dd (114718, BioLegend, 1:100)
Human EpCAM (5447, CST, 1:100)

*Mass cytometry assay:
Anti-Mouse CD45 (30-F11)-89Y (3089005B, Fluidigm, 1:50)
Anti-Mouse CD11b (M1/70)-143Nd (3143015B, Fluidigm, 1:100)
Anti-Mouse Ly-6G (3141008B, Fluidigm, 1:100)
Ly-6C Antibody (HK1.4) - Azide and BSA Free (NBP1-28046, Novus Biologicals, 1:100)
Anti-Mouse I-A/I-E (M5/114.15.2)-209Bi (3209006B, Fluidigm, 1:167)
Anti-Mouse F4/80 (BM8)-146Nd (3146008B, Fluidigm, 1:50)
Anti-Mouse CD86 (GL1)-172Yb (3172016B, Fluidigm, 1:50)
Anti-Mouse CD206/MMR (C068C2)-169Tm (3169021B, Fluidigm, 1:100)
Anti-Mouse CD11c (N418)-142Nd (3142003B, Fluidigm, 1:50)
Anti-mouse CD197 (CCR7), Rat, IgG1, Purified (1200505, Sony Biotechnology, 1:50)
Anti-Mouse CD335/NKp46 (29A1.4)-153Eu (3153006B, Fluidigm, 1:50)
Anti-Mouse CD3e (145-2C11)-152Sm (3152004B, Fluidigm, 1:50)
Anti-Mouse CD4 (RM4-5)-145Nd (3145002B, Fluidigm, 1:50)
Anti-Mouse CD8a (53-6.7)-168Er (3168003B, Fluidigm, 1:50)
Anti-Mouse IFNg (XMG1.2)-165Ho (3165003B, Fluidigm, 1:50)
Arginase 1 antibody [GT5811] (GTX634218, GENETEX, 1:100)
Anti-Mouse iNOS (CXNFT)-161Dy (3161011B, Fluidigm, 1:50)
Anti-Mouse IL-2 (JES6-5H4)-144Nd (3144002B, Fluidigm, 1:50)

*In vitro triculture assay:
InVivoMAb anti-human PD-1 (CD279) (BE0188, BioXCell)

*IHC analysis:
EGFR (ab52894, Abcam, 1:1000)
HER2 (2165, CST, 1:200)
CEACAM6 (#84.7, developed internally; D028-3, MBL, 1:12000)
BRD4 Polyclonal Antibody (A301-985A100, Bethyl, 1:6000)
pSTAT3-Y705 (ab76315, Abcam, 1:100)
pSMAD2-S465-S467 (44-244G, Thermo, 1:100)

pSMAD3-S423-S425 (ab52903, Abcam, 1:100)
collagen I (ab34710, Abcam, 1:50)
Anti-CD8 alpha antibody [EPR21769] (ab217344, Abcam, 1:500)
Anti-CD4 antibody [EPR19514] (ab183685, Abcam, 1:500)
Mouse Granzyme B Antibody (AF1865, R&D, 1:40)
PDGF Receptor α (D1E1E) XP® Rabbit mAb (3174, CST, 1:200)
Monoclonal Anti-Actin, alpha-Smooth Muscle - Alkaline Phosphatase antibody produced in mouse (A5691, Sigma, 1:100)

*IF analysis:
E-Cadherin (AF748, R&D, 1:50; 61081, BD Pharmingen, 1:200)
EGFR (ab52894, Abcam, 1:100)
HER2 (2165, CST, 1:100)
CEACAM6 (ab134074, Abcam, 1:100)
Human IgG (A80-319A, Bethyl, 1:100)

*WB assay:
BRD4 Polyclonal Antibody (A301-985A100, Bethyl, 1:1000)
SMAD2/3 (D7G7) XP® Rabbit mAb (8685, Cell Signaling, 1:1000)
Stat3 (124H6) Mouse mAb (9139, Cell Signaling, 1:1000)
CDK9 (C12F7) Rabbit mAb (2316, Cell Signaling, 1:1000)
Brd2 (D89B4) Rabbit mAb (5848, CST, 1:1000)
GAPDH (14C10) Rabbit mAb (2118, CST, 1:1000)
Phospho-SMAD2 (Ser465/467)/SMAD3 (Ser423/425) (D27F4) Rabbit mAb (8828, CST, 1:1000)
Phospho-Stat3 (Tyr705) (D3A7) XP® Rabbit mAb (9145, CST, 1:2000)
Stat3 (79D7) Rabbit mAb (4904, CST, 1:2000)

*Immunoprecipitation assay:
BRD4 Polyclonal Antibody (A301-985A100, Bethyl, 4 ug/mg lysate)

Validation

Validation data of commercially available antibodies are available on the manufacturer's website. Original CEACAM6 antibody, #84.7, was validated by flow cytometry with CEACAM6-expressing PDAC and CEACAM6-KO PDAC cells. Original HEL3 antibody (control human IgG) was validated by flow cytometry with primary cells and cell lines used in this study.

*ADC prep:
EGFR (cetuximab, Merck)
https://www.merckgroup.com/en/expertise/oncology/oncology-treatments/erbitux.html

HER2 (trastuzumab, Chugai)
https://www.roche.com/solutions/pharma/productid-b6406929-997b-4565-9ffa-cbe89324bbf5

Trop-2 (sacituzumab, purified by EPAT)
Developed internally

HEL3 (purified by KAN, developed internally)
Developed internally

CEACAM6 (#84.7, developed internally)
Developed internally

*Mouse xenograft assay:
InVivoMAb anti-mouse PD-1 (CD279) (BE0146, BioXCell)
Species: Mouse
Application: In vivo blocking of PD-1/PD-L signaling
https://bioxcell.com/invivomab-anti-mouse-pd-1-cd279-be0146

*Monkey biodistribution assay:
EGFR (cetuximab, Merck)
https://www.merckgroup.com/en/expertise/oncology/oncology-treatments/erbitux.html

HER2 (trastuzumab, Chugai)
https://www.roche.com/solutions/pharma/productid-b6406929-997b-4565-9ffa-cbe89324bbf5

HEL3 (purified by KAN, developed internally)
Developed internally

CEACAM6 (#84.7, developed internally)
Developed internally

*Flow cytometry assay:
CEACAM6 (#84.7, developed internally, 10 µg/mL)
Develped internally

CEACAM6 (D028-3, MBL, 10 µg/mL)
Species: Human
Application: WB (10 µg/mL), FC (10 µg/mL)
https://ruo.mbl.co.jp/bio/dtl/A/?pcd=D028-3

Mouse H-2Kd/H-2Dd (114718, BioLegend, 1:100)
Species: Mouse
Application: FC (0.25 µg/10^6 cells)
https://www.biolegend.com/ja-jp/products/pe-cyanine7-anti-mouse-h-2kd-h-2dd-antibody-16131?GroupID=BLG2384

Human EpCAM (5447, CST, 1:100)
Species: Human
Application: IF (1:200-1:800), FC (1:50)
https://www.cellsignal.jp/products/antibody-conjugates/epcam-vu1d9-mouse-mab-alexa-fluor-647-conjugate/5447

*Mass cytometry assay:
Anti-Mouse CD45 (30-F11)-89Y (3089005B, Fluidigm, 1:50)
Species: Mouse
Application: Mass cytometry
https://store.standardbio.com/Cytometry/ConsumablesandReagentsCytometry/MaxparAntibodies/Anti-Mouse%20CD45%20-30-F11-89Y%E2%80%94100%20Tests

Anti-Mouse CD11b (M1/70)-143Nd (3143015B, Fluidigm, 1:100)
Species: Mouse
Application: Mass cytometry
https://store.standardbio.com/Cytometry/ConsumablesandReagentsCytometry/MaxparAntibodies/Anti-Mouse%20CD11b%20-M1-70-143Nd%E2%80%94100%20Tests

Anti-Mouse Ly-6G (1A8)-141Pr (3141008B, Fluidigm, 1:100)
Species: Mouse
Application: Mass cytometry
https://store.standardbio.com/Cytometry/ConsumablesandReagentsCytometry/MaxparAntibodies/Anti-Mouse%20Ly-6G%20-1A8-141Pr%E2%80%94100%20Tests

Ly-6C Antibody (HK1.4) - Azide and BSA Free (NBP1-28046, Novus Biologicals, 1:100)
Species: Mouse
Application: FC (1 µg/10^6 cells)
https://www.novusbio.com/products/ly-6c-antibody-hk14_nbp1-28046#reviews-publications

Anti-Mouse I-A/I-E (M5/114.15.2)-209Bi (3209006B, Fluidigm, 1:167)
Species: Mouse
Application: Mass cytometry
https://store.standardbio.com/Cytometry/ConsumablesandReagentsCytometry/MaxparAntibodies/Anti-Mouse%20I-A-I-E%20-M5-114-15-2-209Bi%E2%80%94100%20Tests

Anti-Mouse F4/80 (BM8)-146Nd (3146008B, Fluidigm, 1:50)
Species: Mouse
Application: Mass cytometry
https://store.standardbio.com/Cytometry/ConsumablesandReagentsCytometry/MaxparAntibodies/Anti-Mouse%20F4-80%20-BM8-146Nd%E2%80%94100%20Tests

Anti-Mouse CD86 (GL1)-172Yb (3172016B, Fluidigm, 1:50)
Species: Mouse
Application: Mass cytometry
https://store.standardbio.com/ccrz__ProductDetails?sku=3172016B&cclcl=en_US

Anti-Mouse CD206/MMR (C068C2)-169Tm (3169021B, Fluidigm, 1:100)
Species: Mouse
Application: Mass cytometry
https://store.standardbio.com/Cytometry/ConsumablesandReagentsCytometry/MaxparAntibodies/Anti-Mouse%20CD206-MMR%20-C068C2-169Tm%E2%80%94100%20Tests

Anti-Mouse CD11c (N418)-142Nd (3142003B, Fluidigm, 1:50)
Species: Mouse
Application: Mass cytometry

https://store.standardbio.com/Cytometry/ConsumablesandReagentsCytometry/MaxparAntibodies/Anti-Mouse%20CD11c%20-N418-142Nd%E2%80%94100%20Tests?cclcl=en_US

Anti-mouse CD197 (CCR7), Rat, IgG1, Purified (1200505, Sony Biotechnology, 1:50)
Species: Mouse
Application: FC (2 μg/10^6 cells)
https://labchem-wako.fujifilm.com/jp/product/detail/W01S531200505.html

Anti-Mouse CD335/NKp46 (29A1.4)-153Eu (3153006B, Fluidigm, 1:50)
Species: Mouse
Application: Mass cytometry
https://store.standardbio.com/ccrz__ProductDetails?refURL=http%3A%2F%2Fstore.fluidigm.com%2FCytometry%2FConsumablesandReagentsCytometry%2FMaxparAntibodies%2FAnti-Mouse%2520CD335-NKp46%2520-29A1-4-153Eu%25E2%2580%2594100%2520Tests&refURL=http%3A%2F%2Fstore.fluidigm.com%2FCytometry%2FConsumablesandReagentsCytometry%2FMaxparAntibodies%2FAnti-Mouse%2520CD335-NKp46%2520-29A1-4-153Eu%25E2%2580%2594100%2520Tests&seoid=Anti-Mouse+CD335-NKp46+-29A1-4-153Eu%E2%80%94100+Tests&sku=3153006B

Anti-Mouse CD3e (145-2C11)-152Sm (3152004B, Fluidigm, 1:50)
Species: Mouse
Application: Mass cytometry
https://store.standardbio.com/Cytometry/ConsumablesandReagentsCytometry/MaxparAntibodies/Anti-Mouse%20CD3e%20-145-2C11-152Sm%E2%80%94100%20Tests?cclcl=en_US

Anti-Mouse CD4 (RM4-5)-145Nd (3145002B, Fluidigm, 1:50)
Species: Mouse
Application: Mass cytometry
https://store.standardbio.com/Cytometry/ConsumablesandReagentsCytometry/MaxparAntibodies/Anti-Mouse%20CD4%20-RM4-5-145Nd%E2%80%94100%20Tests

Anti-Mouse CD8a (53-6.7)-168Er (3168003B, Fluidigm, 1:50)
Species: Mouse
Application: Mass cytometry
https://store.standardbio.com/Cytometry/ConsumablesandReagentsCytometry/MaxparAntibodies/Anti-Mouse%20CD8a%20-53-6-7-168Er%E2%80%94100%20Tests

Anti-Mouse IFNg (XMG1.2)-165Ho (3165003B, Fluidigm, 1:50)
Species: Mouse
Application: Mass cytometry
https://store.standardbio.com/Cytometry/ConsumablesandReagentsCytometry/MaxparAntibodies/Anti-Mouse%20IFNg%20-XMG1-2-165Ho%E2%80%94100%20Tests

Arginase 1 antibody [GT5811] (GTX634218, GENETEX, 1:100)
Species: Human, Mouse, Rat, Monkey
Application: WB (1:500-1:3000), IHC (1:100-1:1000), FC (1:50-1:200), ELISA (1:1000-1:10000), Sandwich ELISA (assay dependent)
https://www.genetex.com/Product/Detail/Arginase-1-antibody-GT5811/GTX634218

Anti-Mouse iNOS (CXNFT)-161Dy (3161011B, Fluidigm, 1:50)
Species: Mouse
Application: Mass cytometry
https://store.standardbio.com/Cytometry/ConsumablesandReagentsCytometry/MaxparAntibodies/Anti-Mouse%20iNOS%20-CXNFT-161Dy%E2%80%94100%20Tests

Anti-Mouse IL-2 (JES6-5H4)-144Nd (3144002B, Fluidigm, 1:50)
Species: Mouse
Application: Mass cytometry
https://store.standardbio.com/Cytometry/ConsumablesandReagentsCytometry/MaxparAntibodies/Anti-Mouse%20IL-2%20-JES6-5H4-144Nd%E2%80%94100%20Tests

*In vitro triculture assay:
InVivoMAb anti-human PD-1 (CD279) (BE0188, BioXCell)
Species: Human
Application: In vitro PD-1 neutralization, In vivo PD-1 blockade in humanized mice
https://bioxcell.com/invivomab-anti-human-pd-1-cd279-be0188

*IHC analysis:
BRD4 Polyclonal Antibody (A301-985A100, Bethyl, 1:6000)
Species: Human, Mouse
Application: WB (1:2000-1:10000), IHC (1:1000-1:5000), IP (2-10 μg/mg lysate)
https://www.fortislife.com/products/primary-antibodies/rabbit-anti-brd4-antibody/BETHYL-A301-985

pSTAT3-Y705 (ab76315, Abcam, 1:100)
Species: Mouse, Human
Application: FC (1:500), WB (1:2000-1:20000), IP (1:20), IHC (1:50-1:100), Dot blot (1:1000), ICC (1:500)
https://www.abcam.co.jp/products/primary-antibodies/stat3-phospho-y705-antibody-ep2147y-ab76315

pSMAD2-S465-S467 (44-244G, Thermo, 1:100)
Species: Human, Mouse
Application: WB (1:1000), IHC (1:20-1:200), ICC (1:250)
https://www.thermofisher.com/antibody/product/Phospho-SMAD2-Ser465-Ser467-Antibody-Polyclonal/44-244G

pSMAD3-S423-S425 (ab52903, Abcam, 1:100)
Species: Mouse, Human
Application: WB (1:2000), ICC (1:100-1:250), IHC (1:100-1:250), Dot blot (1:1000)
https://www.abcam.co.jp/products/primary-antibodies/smad3-phospho-s423--s425-antibody-ep823y-ab52903.html

Collagen I (ab34710, Abcam, 1:50)
Species: Human
Application: IHC (1:15), WB (1:1000-1:10000)
https://www.abcam.co.jp/products/primary-antibodies/collagen-i-antibody-ab34710.html

Anti-CD8 alpha antibody [EPR21769] (ab217344, Abcam, 1:500)
Species: Mouse
Application: IP (1:30), FC (1:500), WB (1:1000), IF (1:500), IHC (1:500)
https://www.abcam.com/en-fi/products/primary-antibodies/anti-cd8-alpha-antibody-epr21769-ab217344

Anti-CD4 antibody [EPR19514] (ab183685, Abcam, 1:500)
Species: Mouse
Application: IP (1:40), WB (1:1000), IF (1:200), IHC (1:1000)
https://www.abcam.com/en-is/products/primary-antibodies/anti-cd4-antibody-epr19514-ab183685

Mouse Granzyme B Antibody (AF1865, R&D, 1:40)
Species: Mouse
Application: IP (25 μg/mL), WB (0.1 μg/mL), IF (5-15 μg/mL), IHC (5-15 μg/mL)
https://www.rndsystems.com/products/mouse-granzyme-b-antibody_af1865

PDGF Receptor α (D1E1E) XP® Rabbit mAb (3174, CST, 1:200)
Species: Human, Mouse
Application: IP (1:50), FC (1:500-1:2000), WB (1:1000), IF (1:1000), IHC (1:500-1:2000)
https://www.cellsignal.jp/products/primary-antibodies/pdgf-receptor-a-d1e1e-xp-rabbit-mab/3174

Monoclonal Anti-Actin, alpha-Smooth Muscle - Alkaline Phosphatase antibody produced in mouse (A5691, Sigma, 1:100)
Species: Human, Mouse, Rat, Chicken, Frog, Canine, Rabbit, Guinea pig, Goat, Bovine, Sheep, Snake
Application: ELISA (assay dependent), IHC (1:20), WB (1:100)
https://www.sigmaaldrich.com/JP/ja/product/sigma/a5691

*IF analysis:
E-Cadherin (AF748, R&D; 1:50)
Species: Human, Mouse
Application: WB (0.5 μg/mL), FC (0.25 μg/10^6 cells), IHC (5-15 μg/mL), ICC (5-15 μg/mL)
https://www.rndsystems.com/products/human-mouse-e-cadherin-antibody_af748

E-Cadherin (61081, BD Pharmingen, 1:200)
Species: Human, Mouse, Rat, Dog
Application: WB (1:10000), IF (1:200), IHC, IP
https://www.bdbiosciences.com/en-us/products/reagents/microscopy-imaging-reagents/immunofluorescence-reagents/purified-mouse-anti-e-cadherin.610182

EGFR (ab52894, Abcam, 1:100)
Species: Mouse, Rat, Human
Application: WB (1:1000-1:10000), IP (1:20), IHC (1:100), ICC (1:250-1:500), ELISA (1:2500), FC (1:20)
https://www.abcam.co.jp/products/primary-antibodies/egfr-antibody-ep38y-ab52894.html

HER2 (2165, CST, 1:100)
Species: Mouse, Human
Application: WB (1:1000), IP (1:100), IHC (1:200-1:800), IF (1:100-1:400), FC (1:200-1:400)
https://www.cellsignal.jp/products/primary-antibodies/her2-erbb2-29d8-rabbit-mab/2165

CEACAM6 (ab134074, Abcam, 1:100)
Species: Human

Application: WB (1:10000-1:50000), IHC (1:100-1:250)
https://www.abcam.co.jp/products/primary-antibodies/ceacam6-antibody-epr4403-ab134074.html

Human IgG (A80-319A, Bethyl, 1:100)
Species: Human
Application: ELISA (1:1000-1:20000), ICC (1:200-1:2000), IHC (1:200-1:2000), WB (1:1000-1:20000)
https://www.fortislife.com/products/secondary-antibodies/goat-anti-human-igg-heavy-and-light-chain-monkey-adsorbed-antibody/BETHYL-A80-319#Applications

*WB assay:
BRD4 Polyclonal Antibody (A301-985A100, Bethyl, 1:1000)
Species: Human, Mouse
Application: WB (1:2000-1:10000), IHC (1:1000-1:5000), IP (2-10 μg/mg lysate)
https://www.fortislife.com/products/primary-antibodies/rabbit-anti-brd4-antibody/BETHYL-A301-985

SMAD2/3 (D7G7) XP® Rabbit mAb (8685, Cell Signaling, 1:1000)
Species: Human, Mouse, Rat, Monkey
Application: WB (1:1000), IP (1:100), IF (1:400-1:800), FC (1:100-1:400), ChIP (1:100), ChIP-seq (1:100)
https://www.cellsignal.jp/products/primary-antibodies/smad2-3-d7g7-xp-rabbit-mab/8685

Stat3 (124H6) Mouse mAb (9139, Cell Signaling, 1:1000)
Species: Human, Mouse, Rat, Monkey
Application: WB (1:1000), Simple Western (1:10-1:50), IP (1:200), IHC (1:300-1:1200), IF (1:800-1:3200), FC (1:50-1:200), ChIP (1:100), CUT&RUN (1:100)
https://www.cellsignal.jp/products/primary-antibodies/stat3-124h6-mouse-mab/9139

CDK9 (C12F7) Rabbit mAb (2316, Cell Signaling, 1:1000)
Species: Human, Mouse, Rat, Hamster, Monkey, Bovine, Dog
Application: WB (1:1000), IP (1:100), IHC (1:100-1:400), IF (1:50-1:100), FC (1:100-1:400), eCLIP (1:200)
https://www.cellsignal.jp/products/primary-antibodies/cdk9-c12f7-rabbit-mab/2316

Brd2 (D89B4) Rabbit mAb (5848, Cell Signaling, 1:1000)
Species: Human, Mouse
Application: WB (1:1000), Simple Western (1:50-1:250), ChIP (1:50), ChIP-seq (1:50), CUT&RUN (1:50)
https://www.cellsignal.jp/products/primary-antibodies/brd2-d89b4-rabbit-mab/5848

GAPDH (14C10) Rabbit mAb (2118, Cell Signaling, 1:1000)
Species: Human, Mouse, Rat, Monkey, Bovine, Dog
Application : WB (1:1000), Simple Western (1:10-1:50), IHC (1:400-1:1600), IF (1:50-1:200), FC (1:100-1:400)
https://www.cellsignal.jp/products/primary-antibodies/gapdh-14c10-rabbit-mab/2118

Phospho-SMAD2 (Ser465/467)/SMAD3 (Ser423/425) (D27F4) Rabbit mAb (8828, Cell Signaling, 1:1000)
Species: Human, Mouse, Rat, Monkey
Application: WB (1:1000)
https://www.cellsignal.jp/products/primary-antibodies/phospho-smad2-ser465-467-smad3-ser423-425-d27f4-rabbit-mab/8828

Phospho-Stat3 (Tyr705) (D3A7) XP® Rabbit mAb (9145, Cell Signaling, 1:2000)
Species: Human, Mouse, Rat, Monkey
Application: WB (1:2000), Simple Western (1:10-1:50), IP (1:100), IHC Leica Bond (1:100-1:400), IHC (1:100-1:400), IF (1:100-1:200), FC (1:100-1:400), ChIP (1:100), ChIP-seq (1:100)
https://www.cellsignal.jp/products/primary-antibodies/phospho-stat3-tyr705-d3a7-xp-rabbit-mab/9145

Stat3 (79D7) Rabbit mAb (4904, Cell Signaling, 1:2000)
Species: Human, Mouse, Rat, Monkey
Application: WB (1:2000), IP (1:100), ChIP (1:50)
https://www.cellsignal.jp/products/primary-antibodies/stat3-79d7-rabbit-mab/4904

*Immunoprecipitation assay:
BRD4 Polyclonal Antibody (A301-985A100, Bethyl, 4 μg/mg lysate)
Species: Human, Mouse
Application: WB (1:2000-1:10000), IHC (1:1000-1:5000), IP (2-10 μg/mg lysate)
https://www.fortislife.com/products/primary-antibodies/rabbit-anti-brd4-antibody/BETHYL-A301-985

Eukaryotic cell lines

Policy information about cell lines and Sex and Gender in Research

Cell line source(s)

Human PDAC cell lines, HPAF-II and AsPC-1, and HEK293 cells were purchased from ATCC. Mouse PDAC cell line, Pan02, was purchased from NIH. Primary human PDAC cells, KYK models and PC models, were obtained from KAN Research Institute and

	The Tsukuba Human Tissue Biobank Center. Human stellate cells, LSC and PSC, and pulmonary alveolar epithelial cells (HPAEpiC) were purchased from ScienCell Research Laboratories. Human PBMCs, hematopoietic progenitor cells (HPC) and small airway epithelial cells (HSAEC) were purchased from Lonza. Mouse cancer-associated fibroblasts (CAF) were isolated from PDAC-PDX tumor.
Authentication	Commercially available cells were not authenticated. Primary human PDAC cells were examined by sequencing oncogenes. Mouse CAFs were examined by fibroblast marker expression.
Mycoplasma contamination	All cell lines were tested negative for mycoplasma contamination. Human PDAC cell lines were routinely tested by ourselves, and commercially available cells were tested by vendors.
Commonly misidentified lines (See ICLAC register)	None.

Animals and other research organisms

Policy information about studies involving animals; ARRIVE guidelines recommended for reporting animal research, and Sex and Gender in Research

Laboratory animals	4-6 weeks old female NOD-SCID, NSG and C57BL/6 mice were purchased from Charles River and housed in 23±3 °C, a humidity of 55 ±15%, and a 12 hours dark/light cycle. 3-4 years old cynomolgus monkeys were purchased from Tian Hu Cambodia Animal Breeding Research Center Ltd. and housed in 26±3 °C, a humidity of 50±20%, and a 12 hours dark/light cycle at the facility of Shin Nihon Kagaku.
Wild animals	This study did not involve sample from wild animals.
Reporting on sex	Only female mice were used in this study, considering the effect of fighting between male mice on the evaluation of drug efficacy.
Field-collected samples	This study did not involve filed-collected sample.
Ethics oversight	All the animal experiments were conducted in accordance with the Institutional Animal Care and Use Committee guidelines of Eisai Co., Ltd. and Shin Nihon Kagaku, Ltd. (animal study protocols of Eisai v18, SBL038-158)

Note that full information on the approval of the study protocol must also be provided in the manuscript.

Flow Cytometry

Plots

Confirm that:

- [x] The axis labels state the marker and fluorochrome used (e.g. CD4-FITC).
- [x] The axis scales are clearly visible. Include numbers along axes only for bottom left plot of group (a 'group' is an analysis of identical markers).
- [x] All plots are contour plots with outliers or pseudocolor plots.
- [x] A numerical value for number of cells or percentage (with statistics) is provided.

Methodology

Sample preparation	For 2D-cultured cells, cells were harvested by TrypLE Select (Thermo). For xenograft models, tumors were extirpated from mice and digested with a Tumor Dissociation Kit and gentleMACS Dissociator (Miltenyi Biotec). After being washed with PBS containing 2 mM EDTA and 0.3% BSA, the cells were preincubated with the human Fc receptor binding inhibitor Clear Back (MBL). The cells were then incubated with antibodies mentioned above.
Instrument	LSRFortessa flow cytometry system
Software	FlowJo software (v10.8.1)
Cell population abundance	The study did not include cell sorting.
Gating strategy	Dead cells and small debris were eliminated in FSC/SSC plots, and then alive cells were separated as DAPI negative cells. Human PDAC cells were examined in human EpCAM positive and mouse MHC negative cells.

- [x] Tick this box to confirm that a figure exemplifying the gating strategy is provided in the Supplementary Information.
